# Der Zusammenhang zwischen schulischer Tabakkontrolle und der wahrgenommenen Raucherprävalenz Jugendlicher

**DOI:** 10.1007/s00103-020-03261-1

**Published:** 2020-12-07

**Authors:** Martin Mlinarić, Sebastian Günther, Irene Moor, Kristina Winter, Laura Hoffmann, Matthias Richter

**Affiliations:** Institut für Medizinische Soziologie, Medizinische Fakultät, Martin-Luther-Universität Halle-Wittenberg, Magdeburger Str. 8, 06112 Halle (Saale), Deutschland

**Keywords:** Schulische Tabakkontrolle, Rauchen, Tabak, Soziale Ungleichheit, Prävention, School tobacco policies, Smoking, Tobacco, Social inequalities, Prevention

## Abstract

**Hintergrund:**

Für Deutschland sind kaum Daten zur Implementierung schulischer Tabakkontrolle bekannt, die sowohl die Perspektive von Lehrer- als auch von Schüler*innen einbeziehen. Ziel der Studie ist es, den Zusammenhang zwischen implementierter schulischer Tabakkontrollpolitik und der wahrgenommenen Raucherprävalenz auf den Ebenen des Schulpersonals und der Jugendlichen zu untersuchen.

**Methoden:**

Die wiederholte Querschnittsstudie (2013 und 2017) basiert auf gepoolten Angaben von 13- bis 17-jährigen Jugendlichen (*N* = 2393) und Schulpersonal (*N* = 85) aus 25 Schulen in der westdeutschen Metropolregion Hannover. In linearen Regressionsmodellen werden durchschnittlich marginale Effekte (AMEs) mit 95 %-Konfidenzintervallen (KI95 %) und robuste Standardfehler für die wahrgenommenen Tabakprävalenzen getrennt für die von Lehrer- und Schüler*innen bewertete schulische Tabakkontrollpolitik (Skala 0–6) unter Kontrolle soziodemografischer, schul- und rauchspezifischer Variablen berichtet.

**Ergebnisse:**

Die Jugendlichen nehmen durchschnittlich eine Raucherprävalenz von 30 % ($\overline{x}=30,2$; s: 24,0) für ihre Schule wahr. Eine strikte schulische Tabakkontrolle ist konsistent mit einer geringeren schulischen Raucherprävalenz assoziiert, sowohl aus Sicht von Lehrer- (AME: −3,54 KI95 % −6,49 bis −0,58) als auch der Schüler*innen (AME: −1,69 KI95 % −2,52 bis −0,86). Die Anzahl rauchender Freunde (z. B. „die meisten“ +14 %: AME: 14,13 KI95 % 10,46–17,80) und der Schultyp sind die relevantesten Determinanten für eine hohe schulische Raucherprävalenz. Nichtgymnasiale Schulformen berichten eine 15 % (AME: 15,03 KI95 % 10,13–19,93) höhere Raucherprävalenz im Vergleich zu Gymnasien.

**Diskussion:**

Progressive schulische Tabakkontrollpolitiken sollten den Fokus auf nichtgymnasiale Schulformen, Risikogruppen und jene Schulen legen, die Tabakkontrolle nicht strikt durchsetzen.

**Zusatzmaterial online:**

Zusätzliche Informationen sind in der Online-Version dieses Artikels (10.1007/s00103-020-03261-1) enthalten.

## Abstract (en)

**Background:**

In the German context, there is hardly any quantitative data about the implementation of school tobacco polices that include the perspective of both teachers and students. The aim of the study is to investigate the associations between implemented school tobacco policies and the perceived prevalence of smoking at the level of school staff and adolescents.

**Methods:**

The repeated cross-sectional study (2013 and 2017) is based on pooled responses of 13- to 17-year-old adolescents (*N* = 2393) and school staff (*N* = 85) from 25 schools located in the West German metropolitan region of Hanover. In linear regression models, average marginal effects (AMEs) with 95% confidence intervals (CI95%) and robust standard errors for perceived tobacco prevalence are reported separately for school tobacco policies assessed by teachers and students (scale 0–6). All models were controlled for sociodemographic, school-, and smoking-specific covariates.

**Results:**

On average, adolescents perceive a smoking prevalence of 30% ($\overline{x}=30.2$; s: 24.0) for their school. A comprehensive school tobacco policy is consistently associated with lower school smoking prevalence both from the point of view of teachers (AME: −3.54 CI95% −6.49 to −0.58) and students (AME: −1.69 CI95% −2.52 to −0.86). The number of smoking friends (e.g., “most of them are smokers” +14%: AME: 14.13 CI95% 10.46 to 17.80) and the type of school are the most relevant determinants of a high school smoking prevalence. School types with a nonacademic track report a 15% (AME: 15.03 CI95% 10.13 to 19.93) higher prevalence compared to grammar schools.

**Discussion:**

Progressive school tobacco control policies should focus more on school types with nonacademic tracks, certain groups at risk, and those schools that do not strictly enforce school tobacco policies.

## Hintergrund

In Deutschland gehen laut Deutschem Krebsforschungszentrum 13 % aller Todes- und 19 % der Krebsfälle auf Langzeitfolgewirkungen des Rauchens von Tabakprodukten zurück [1, 2]. Die im Jahr 2003 von der Weltgesundheitsorganisation (WHO) verabschiedete „Rahmenvereinbarung über Tabakkontrolle“ [3] bewirkte, dass Tabakkontrollpolitiken – wie etwa Steuererhöhungen, öffentliche Rauchverbote und bildgestützte Warnhinweise – sukzessiv auf nationaler Ebene implementiert worden sind [4]. Nach aktuellster Studienlage haben diese Interventionen innerhalb der Europäischen Union (EU) sowie in Mittel- und Hocheinkommensländern zu einer Reduzierung der Raucherprävalenzen beigetragen [5, 6]. Soziale Ungleichheiten nach sozioökonomischem Status (SES) sind im Rauchen jedoch sowohl im Jugend- [7–9] als auch Erwachsenenalter persistent [10, 11].

## Forschungsstand zur Implementierung schulischer Tabakkontrolle

Nationale und internationale Studien belegen für Hocheinkommensländer einen dominanten sozialen Gradienten in der Tabakexposition von Heranwachsenden im häuslich-familiären Kontext [12–14]. Studien zu sozialen Ungleichheiten im jugendlichen Rauchen dokumentieren, dass der Rauchstatus elternhaus-, freundes- und schulformabhängig ist [15, 16]. Soziale Ungleichheiten und Trends im Rauchverhalten werden häufig im Hinblick auf selbstberichtete Raucherprävalenzen sozialepidemiologisch untersucht [17, 18]. Vor dem Hintergrund regressiver Raucherprävalenzen im Jugendalter in Deutschland [19] bleibt es weitestgehend unbeantwortet, inwiefern sich das von Jugendlichen empfundene Ausmaß der Raucherprävalenz in einem zentralen Sozialisationskontext wie der Schule darstellt.

Quantitative Studien konnten aufzeigen, dass schulische Tabakkontrolle nicht unbedingt mit dem eigenen Rauchstatus assoziiert ist [16] oder stigmatisierend wirkt [20], es jedoch einen deutlichen linearen Zusammenhang zwischen tatsächlicher (selbstberichteter) Tabakprävalenz im Sinne des eigenen Rauchens und der hohen Sichtbarkeit des Rauchens an der Schule gibt [21]. Die wahrgenommene Prävalenz des Rauchens im Jugendalter ist von herausragender Bedeutung für die Tabakprävention, da eine hohe Wahrnehmung des Rauchens in der unmittelbaren Lebenswelt – wie der Schule – nicht nur mit Rauchinitiierung und positiven Einstellungen gegenüber Tabakrauchen [22], sondern auch leichterem Zugang zu Zigaretten [23] und einer nachgiebigen Umsetzung von schulischen Rauchverboten assoziiert ist [22, 24]. Der lokale [25] und der schulische [24, 26] Kontext von Tabakkontrolle sind für Ungleichheiten in der Sichtbarkeit [22] des Rauchens als sozialräumliche Sozialisationsorte zentral, wobei sie stadt- oder länderübergreifend variieren [27, 28].

Die Evidenz zur Effektivität rauchfreier Schulen ist hinsichtlich der Prävention jugendlichen Rauchens fragmentarisch [29]. Laut einer systematischen Übersichtsarbeit kommt es vor allem auf den Modus der Implementierung einer rauchfreien Schule an [24]. Demnach sollten Rauchverbote im Schulkontext prinzipiell sowohl für Schüler- als auch Lehrer*innen gelten, um eine rauchfreie Schule zu implementieren [24, 30]. Partielle Rauchverbote, geringe Priorisierung, eine Attitüde „des blinden Auges“[31] vonseiten der Lehrerschaft, mangelnde zeitliche Ressourcen oder rauchendes Schulpersonal sind jedoch wesentliche Barrieren in der Umsetzung von Tabakkontrolle im Schulkontext [32]. Uneinheitliche Regeln – wie etwa das Zugestehen des Rauchens für das Schulpersonal – können zudem Widerstand und positive Einstellungen gegenüber dem Rauchen aufseiten der Jugendlichen fördern [24, 27, 30]. Lehrer*innen wird die Umsetzung der schulischen Tabakkontrolle meist aufgetragen, jedoch kann das Schulpersonal aus weltanschaulichen (z. B. libertäre Autonomievorstellungen) oder auch pädagogischen Erwägungen (z. B. Fokus auf ganzheitliche Persönlichkeitsentwicklung der Jugendlichen) davon absehen, Verstöße gegen die Regeln zum Rauchen auf dem Schulgelände und in dessen Umgebung strikt zu sanktionieren [33].

## Zielstellung und Forschungsfragen

Für den deutschen Kontext gibt es bisher kaum quantitative Daten zur Implementierung schulischer Tabakkontrolle, die sowohl die Perspektive von Lehrer- als auch die der Schüler*innen einbeziehen. Ziel der vorliegenden Studie ist es, die Assoziation zwischen implementierter schulischer Tabakkontrollpolitik als Expositionsgröße und der wahrgenommenen Prävalenz des Rauchens im Hinblick auf die Realisierung einer rauchfreien Schule zu untersuchen. Diese Zusammenhänge werden auf den Ebenen von Lehrer- und Schülerschaft getrennt anhand einer wiederholten Querschnittsstudie (2013 und 2017) quantitativ untersucht. Aus der existierenden internationalen Forschungslage zu jugendlichen Rauchkontexten und schulischer Tabakkontrolle lassen sich 3 leitende Forschungsfragen für die vorliegende Untersuchung zu rauchfreien Schulen ableiten:

1. Inwiefern korreliert die von Schüler- und Lehrer*innen berichtete schulische Tabakkontrolle mit der wahrgenommenen schulischen Prävalenz des Rauchens?
2. Bestehen im Kontext „rauchfreier“ Schulen für die Heranwachsenden soziale Ungleichheiten im Hinblick auf die wahrgenommene Raucherprävalenz?
3. Wie groß ist das Ausmaß möglicher sozialer oder schulspezifischer Ungleichheitskorrelate hinsichtlich der Tabakprävalenz?

## Methodik

Zwei Befragungswellen der Jahre 2013 (SILNE) und 2017 (SILNE-R) wurden mit Unterstützung der Europäischen Kommission zum Thema sozialer Ungleichheiten im Rauchen europäischer Jugendlicher erhoben. Standardisierte Fragebögen wurden in Klassenräumen oder Mensen schriftlich ausgefüllt. Der deutschsprachige Lehrer- und Schülerfragebogen befindet sich im Online-Zusatzmaterial zu diesem Beitrag. Insgesamt wurden Befragungen in 25 Schulen in Hannover und Umgebung (u. a. Lehrte, Celle) durchgeführt [34]. Die Stadt/Region Hannover kann im Hinblick auf sozialstrukturelle Daten (u. a. Arbeitslosenquote, Personen mit Migrationshintergrund) als adäquate Repräsentanz einer durchschnittlichen (west-)deutschen Großstadt verstanden werden [34].

## Stichprobe

Die vorliegende wiederholte Querschnittsstudie umfasste 2 Stichproben in den Jahren 2013 und 2017 mit Lehrer- (*n* = 122) und Schüler*innen (*n* = 2976) aus insgesamt 25 teilnehmenden Schulen. Bei den gepoolten Schulbefragungen handelte es sich um eine geschichtete und quotierte Zufallsstichprobe. Die jeweiligen verantwortlichen Ansprechpartner*innen der 8. und 9. Klassen aus über 60 öffentlichen und privaten Schulen waren pro Erhebungswelle in Hannover und Umgebung angeschrieben worden. Pro Erhebungswelle war eine Fallzahl von mindestens 1400 Befragten anvisiert worden. Die Studiengruppe war aufgrund der bekannten sozialen Ungleichheiten im jugendlichen Rauchverhalten stets darauf bedacht, ein möglichst ausgeglichenes Verhältnis zwischen gymnasialen und nichtgymnasialen Schulformen herzustellen.

Nach Ausschluss fehlender Werte (*n* = 620) beinhaltete die Stichprobe, neben dem befragten Schulpersonal (*N* = 85), Jugendliche (*N* = 2393) der 8. und 9. Schuljahrgänge im Alter von mindestens 13 bis maximal 19 Jahren aus 11 Haupt‑, Real- und integrierten Gesamtschulen, einer Privatschule und 8 Gymnasien. Die Rücklaufquote auf Schülerebene lag in beiden Erhebungswellen bei 66 % [34]. Datenschutzrechtliche Vorgaben sowie die notwendige aktive elterliche Einwilligung beeinträchtigten eine höhere Teilnahmebereitschaft.

## Abhängige Variable

Die Zielgröße der geschätzten Prävalenz des Rauchens bezieht sich in der vorliegenden Studie auf das von Schüler*innen berichtete Ausmaß des wahrgenommenen jugendlichen Rauchens von Tabakprodukten (Zigaretten, selbst gedrehte Zigaretten, Zigarren, Zigarillos) an ihrer jeweiligen Schule [21]. Das Rauchen von E‑Produkten, Shisha oder Cannabis ist nicht eingeschlossen. Hinsichtlich der wahrgenommenen Raucherprävalenz wurden die Schüler*innen auf einer numerischen Analogskala, die von 0 bis 100 (%) reichte, gefragt: „Was schätzt Du? Wie viele Leute in Deinem Alter rauchen in etwa an Deiner Schule?“ [21]. Die in Prozentpunkten geschätzte schulische Raucherprävalenz (0 %, 5 %, 10 %, 20 %, 30 %, 40 %, 50 %, 60 %, 70 %, 80 %, 90 %, 100 %) wurde zum Zwecke der linearen Regression als metrische Variable beibehalten [21].

## Unabhängige Variable

Die unabhängige Variable und Expositionsgröße umschreibt die Qualität und Durchsetzungspraktiken eines Maßnahmenbündels schulischer Tabakkontrollmaßnahmen und wurde in Anlehnung an die empirischen Arbeiten von Schreuders [27, 31], Mélard [26] und Kuipers et al. [16] auf mehreren Ebenen operationalisiert [26]. Hierbei wurde die Qualität schulischer Tabakkontrolle sowohl aufseiten der Lehrer- als auch der Schüler*innen anhand verschiedenster Implementierungsindikatoren (u. a. Regelung und Durchsetzung von Rauchverboten, Zugang zu Tabak in der Schulumwelt und Rolle des Themas im Lehrplan) abgefragt und jeweils auf einer metrischen Summenskala von 0 (schwach) bis 6 (strikt) bewertet. Aufseiten des Lehrpersonals wurde der allgemeine schulische Kontext im Hinblick auf die tabakbezogene Umwelt (Rauchverbote, Zugang zu Tabak und Tabak im Schulcurriculum) erfasst, wohingegen die Angaben der Schüler*innen die alltägliche Implementierung der schulischen Rauchverbote für Lehrer- und Schülerschaft maßen. Die präzise Operationalisierung auf Basis von 15 Fragen zur schulischen Tabakkontrolle ist der Syntax im Online-Zusatzmaterial zu entnehmen.

Aufseiten des Schulpersonals (*N* = 85) wurde ermittelt, ob Rauchverbote gemäß niedersächsischem Schulerlass (vom 01.08.2005) bestehen, Schüler*innen im letzten halben Jahr dagegen verstoßen haben, Zigaretten oder Tabakwaren in weniger als 100 m Entfernung gekauft werden können und Unterrichtsstunden zu den Folgen des Rauchens Teil des Schulcurriculums sind (jeweils Ja = 0,5, Nein = 0). Hinsichtlich der Gestaltung der Rauchverbote wurde vertiefend erhoben, ob jene während der Schulstunden für a.) Jugendliche und/oder b.) das Schulpersonal im Schulgebäude, auf dem Schulgelände einschließlich Park- und Sportplätzen sowie außerhalb des Schulgeländes oder auf Schulveranstaltungen bestehen (jeweils Ja = 0,5; Nein = 0). Ferner wurde nach Raucherzonen, -inseln bzw. -räumen für Jugendliche und das Schulpersonal (jeweils Ja = 0,5, Nein = 0) gefragt [27, 31]. Die Mittelwerte der Lehrersummenskala wurden stratifiziert nach Schule berechnet und jeweils den befragten Jugendlichen der 25 Schulen im Datensatz global zugewiesen [27, 31].

Aufseiten der von Schüler*innen (*N* = 2393) berichteten schulischen Tabakkontrolle wurde in Rechnung gestellt, ob die Jugendlichen auf dem Schulgelände rauchen dürfen („Nein, die Regel wird strikt durchgesetzt“ = 2, „Nein, die Regel wird aber nicht strikt durchgesetzt“ = 1, „Ja, in bestimmten Bereichen“ = 0,5, „Ja, überall auf dem Schulgelände“ = 0, „weiß ich nicht“ = 0). Die Antwortoption „weiß ich nicht“ wurde für die Analyse beibehalten und erhielt 0 Punkte, da um das Rauchverbot nicht wissende Schüler*innen nicht von der schulischen Tabakkontrolle profitieren [27, 31]. Ferner wurde die konkrete Durchsetzung für sowohl sichtbar rauchende a.) Schüler- als auch b.) Lehrer*innen auf dem Schulgelände erhoben, indem die heranwachsenden Befragten jeweils mit „nie“ (= 2), „manchmal“ (= 1), „oft“ (= 0,5) oder „immer“ (= 0) antworten konnten [27, 31].

## Soziodemografische Kontrollvariablen

Mit 1236 Mädchen und 1157 Jungen basiert die vorliegende Stichprobe auf der Altersgruppe der 13- bis 17-jährigen Jugendlichen [15, 16]. Jener Fokus lässt sich damit rechtfertigen, als dass der Übergang vom Experimentieren hin zum regulären Rauchen in dieser Periode des Jugendalters stattfindet [35].

Der Migrationsstatus wurde in ein- und zweiseitige Migrant*innen kategorisiert [36]. Hierbei wurden Antworten auf die Fragen: „In welchem Land ist Deine Mutter geboren?“ und „In welchem Land ist Dein Vater geboren?“, zugrunde gelegt. Ein einseitiger Migrationsstatus wurde zugewiesen, wenn mindestens ein Elternteil im Ausland geboren wurde, und zweiseitige Migration, wenn beide Eltern in einem anderen Staat – mit Ausnahme der DDR – geboren wurden.

## Sozioökonomischer Status, Schulform und schulische Leistungen

Bildungsindikatoren der Eltern sind mit gesundheitlichen Ungleichheiten ihrer Kinder, jugendlichem Tabakkonsum und der schulformabhängigen Exposition gegenüber dem Rauchen assoziiert [7, 8, 16]. Neben dem familiären Wohlstand ist das Bildungsniveau der Eltern ein wesentlicher Bestimmungsfaktor für den SES der Familie und den jugendlichen Rauchstatus [7, 37]. Der SES der Eltern wurde als hohe, mittlere, niedrige und unbekannte mütterliche und väterliche Bildung erfasst [16].

Empirische Befunde verdeutlichen, dass Schulformen mit gesundheitlichen Ungleichheiten im Jugendalter korrelieren [38]. Die Schulform ist in der vorliegenden Analyse ein möglicher Confounder (Störvariable), da sie sowohl mit dem Niveau der schulischen Tabakkontrolle als auch der wahrgenommenen Tabakprävalenz assoziiert ist. Die Schulform der befragten Jugendlichen ist ferner ein Proxy für den individuellen SES und wurde nominal codiert [39]. Die 25 Schulen wurden in gymnasiale und nichtgymnasiale Schulformen dichotomisiert.

Schulische Leistungen sind ferner ein möglicher Indikator für späteren beruflichen Erfolg und den zukünftigen Sozialstatus [40]. Schulleistungen sind ebenso mit dem eigenen Rauchstatus [41] sowie der wahrgenommenen Raucherprävalenz an der Schule [21] assoziiert. Schüler*innen mit schlechten Schulnoten sind häufiger tägliche Raucher*innen [41] und nehmen allgemein eine höhere Tabakprävalenz in ihrer Umgebung wahr als leistungsstarke Klassenkamerad*innen [21]. Die Schüler*innen wurden nach der Schulnote gefragt, die ihre schulische Leistung im letzten Schuljahr am ehesten beschreibt. Die schulischen Leistungen wurden ordinal und damit als hoch (Note 2 oder besser), mittel (Note 3) oder niedrig (Note 4 oder schlechter) kategorisiert.

## Rauchspezifische Mediatoren

Das eigene und elterliche Rauchen oder auch das Rauchen der Freunde vermittelt, dass Jugendliche eine erhöhte Tabakprävalenz in ihrer sozialen Umgebung wahrnehmen [16, 21, 27, 36]. Der individuelle Rauchstatus der Jugendlichen wurde als „wöchentlich“ („mindestens 1–2 Zigaretten wöchentlich“), „gelegentlich“ (≤1–2 Zigaretten wöchentlich) sowie „niemals“ kategorisiert. Das elterliche Rauchen wurde in 4 Gruppen eingeteilt: „keine“, „Ex-Raucher“, „ein“ und „zwei oder mehr“ rauchende Elternteile. Die soziale Nähe zu rauchenden Freunden wurde erfasst, indem gefragt wurde, wie viele der „besten und engsten Freunde“ rauchen, worauf die Befragten mit „keiner“, „einige“, „die meisten“ und „alle“ antworten konnten.

## Statistische Analyse

In der statistischen Analyse wurden multivariate lineare Regressionsmodelle hinsichtlich der wahrgenommenen Raucherprävalenz auf der jeweilig von Lehrer- (Modell 1) und Schüler*innen (Modell 2) getrennt berichteten schulischen Tabakkontrolle berechnet. Die Assoziationen wurden für die Erhebungsjahre, soziodemografische und ökonomische Merkmale sowie Aufnahme von schul- und rauchspezifischen Mediatoren respektive Störvariablen (Schulform) kontrolliert.

In den Modellen werden die durchschnittlich marginalen Effekte (AMEs) mit 95 %-Konfidenzintervallen (KI) für die wahrgenommene Prävalenz des Rauchens berichtet. Die AMEs können im Hinblick auf die berichteten Raucherprävalenzen als Prozentunterschiede interpretiert und im Gegensatz zu Risikomaßen wie Odds Ratios (ORs) weder im Ausmaß überschätzt noch durch die unbeobachtete Heterogenität verzerrt werden [42]. Für alle Berechnungen wurden robuste Standardfehler (RSE) berechnet, um der in 25 Schulen geclusterten Datenstruktur gerecht zu werden. Sensitivitätsanalysen wurden zudem mit ordinal statt metrisch skalierten unabhängigen Variablen sowie unter Einführung eines Random Intercept für die Schulzugehörigkeit vorgenommen, wobei die Ergebnisse in dieselbe Richtung verwiesen. Datenaufbereitung und -bereinigung sowie sämtliche Berechnungen wurden mit STATA 14.0 (*StataCorp LLC, College Station, USA*) durchgeführt.

## Ergebnisse

Die deskriptiven Charakteristika der Studienpopulation sind in Tab. 1 aufgeführt. Das Durchschnittsalter der Heranwachsenden aus der Stichprobe liegt bei 14 Jahren ($\overline{x}=14,4$; s: 0,9). Durchschnittlich nehmen die Jugendlichen eine Raucherprävalenz von 30 % ($\overline{x}=30,2$; s: 24,0) für ihre Schule wahr, wobei sich die wahrgenommene Prävalenz zwischen 2013 ($\overline{x}=40,3$; s: 23,5) und 2017 ($\overline{x}=19,4$; s: 19,3) halbiert hat.

**Table 1:** 

Erhebungsjahr		2013	2017	Total	Fehlende Werte
*N* (Lehrer*innen)	**–**	*N* = 53	*N* = 32	*N* = 85	*n* = 37 ^a^
*N* (Schüler*innen)	**–**	*N* = 1235	*N* = 1158	*N* = 2393	*n* = 583
Alter Schüler*innen	**–**	$\overline{x}(s)$	$\overline{x}(s)$	$\overline{x}(s)$	–
		14,7 (0,9)	14,2 (0,9)	14,4 (0,9)	*n* = 42
**Abhängige Variable**					
*Schulische Raucherprävalenz*	Skala 0–100 %	$\overline{x}(s)$	$\overline{x}(s)$	$\overline{x}(s)$	–
		40,3 (23,5)	19,4 (19,3)	30,2 (24,0)	*n* = 77
**Unabhängige Variable**					
*Schulische Tabakkontrolle* *(Skalenwert 0–6)*	–	$\overline{x}(s)$	$\overline{x}(s)$	$\overline{x}(s)$	–
	Lehrer*innen	4,7 (0,4)	4,6 (0,6)	4,7 (0,5)	*n* = 37^a^
	Schüler*innen	3,2 (1,3)	4,0 (1,4)	3,6 (1,4)	*n* = 109^b^
**Kontroll- und Mediatorvariablen**					
**–**	**–**	**%**	**%**	**%**	**–**
*Alter*	16 oder älter	16,5	7,0	11,9	*n* = 42
	15	37,3	26,0	31,8	
	14 oder jünger	46,2	67,0	56,3	
*Geschlecht*	Weiblich	51,2	52,2	51,6	*n* = 43
	Männlich	48,8	47,8	48,4	
*Migrationshintergrund*	Zweiseitig	28,1	23,5	25,9	*n* = 70
	Einseitig	11,5	16,1	13,7	
	Keiner	60,4	60,4	60,4	
*Bildung* *Mutter*	Unbekannt	21,0	21,3	21,2	*n* = 136
	Niedrig	12,2	5,9	9,2	
	Mittel	38,7	32,6	35,7	
	Hoch	28,1	40,2	34,0	
*Bildung* *Vater*	Unbekannt	23,7	25,0	24,3	*n* = 130
	Niedrig	12,7	10,0	11,4	
	Mittel	33,2	25,1	29,3	
	Hoch	30,4	39,9	35,0	
*Schulform*	Nichtgymnasial	57,3	34,2	46,1	*n* =0
	Gymnasial	42,7	65,8	53,9	
*Schulische* *Leistungen*	Niedrig	19,4	12,8	16,2	*n* = 135
	Mittel	48,5	46,4	47,5	
	Hoch	32,1	40,9	36,4	
*Rauchstatus*	Wöchentlich	10,9	4,3	7,7	*n* = 45
	Gelegentlich	32,5	18,3	25,6	
	Niemals	56,7	77,4	66,7	
*Elterliches* *Rauchen*	≥2 Raucher	17,4	14,9	16,2	*n* = 250
	1 Raucher	31,6	25,7	28,8	
	Ex-Raucher	23,4	18,2	20,9	
	Keiner	27,6	41,2	34,2	
*Rauchende* *Freunde*	Einige	38,1	27,3	32,9	*n* = 95
	Die meisten	10,3	4,0	7,2	
	Alle	2,1	0,5	1,3	
	Keiner	49,6	68,2	58,6	

$$\overline{x}$$ Mittelwert, *(s)* Standardabweichung

^a^Spezifikation der fehlenden Werte im Lehrerfragebogen: Rauchverbote gemäß niedersächsischem Schulerlass (*n* = 2), Verstöße im letzten halben Jahr (*n* = 6), Zigaretten oder Tabakwaren in weniger als 100 m Entfernung erhältlich (*n* = 6), Unterrichtsstunden zu den Folgen des Rauchens Teil des Schulcurriculums (*n* = 17), Rauchverbote gelten während der Schulstunden für Jugendliche (*n* = 2) und das Schulpersonal (*n* = 7) im Schulgebäude, auf dem Schulgelände einschließlich Park- und Sportplätzen für Jugendliche (*n* = 2) und das Schulpersonal (*n* = 11), außerhalb des Schulgeländes oder auf Schulveranstaltungen für Jugendliche (*n* = 7) und das Schulpersonal (*n* = 20), Raucherzonen, -inseln bzw. -räume für Jugendliche (*n* = 4) und das Schulpersonal (*n* = 5)

^b^Spezifikation der fehlenden Werte im Schülerfragebogen: Wahrnehmung der Durchsetzung des schulischen Rauchverbots (*n* = 69), Wahrnehmung sichtbar rauchender Schüler- (*n* = 65) und Lehrer*innen (*n* = 83)

Die von Lehrer*innen berichtete schulische Tabakkontrolle wird zu beiden Erhebungswellen vom Schulpersonal nahezu identisch (2013: $\overline{x}=4,7$; s: 0,4/2017: $\overline{x}=4,6$; s: 0,6) und im Vergleich zu den jugendlichen Befragten besser bewertet, wobei der Anteil strikter schulischer Tabakkontrolle zwischen 2013 ($\overline{x}=3,2$; s: 1,3) und 2017 ($\overline{x}=4,0$; s: 1,4) bei der befragten Schülerschaft um 0,8 Punkte zunimmt.

Die Mehrheit der Jugendlichen raucht nicht (67 %; Tab. 1). Der Anteil der wöchentlich Rauchenden hat sich in der Stichprobe zwischen beiden Erhebungswellen sogar von 11 % auf 4 % reduziert. Dies korrespondiert mit der wahrgenommenen sinkenden schulischen Raucherprävalenz um etwa 14 % vonseiten der Lehrer- bzw. um 12 % aufseiten der Schülerschaft (Tab. 2 und 3). Eine Minderheit der Schüler*innen (9 %) berichtet, mehrheitlich rauchende Freunde zu haben (Tab. 1). Fast die Hälfte (45 %) der befragten Jugendlichen berichtet jedoch, kein rauchfreies Familienumfeld und damit mindestens einen rauchenden Elternteil zu haben.

**Table 2:** 

	*N* = 2393	Modell 1: Lehrer*innen	
		AMEs (KI95 %)^a^	RSE^b^
**Unabhängige Variable (Exposition)**			
Schulische Tabakkontrolle	(Skalenwert 0–6) Lehrer*innen	−3,54 (−6,49–−0,58)*	1,43
**Kontroll- und Mediatorvariablen**			
*Erhebungsjahr*	2017	−13,51 (−16,72–−10,30)***	1,55
	2013	REF	
*Alter*	16 oder älter	7,44 (3,26–11,61)**	2,01
	15	4,41 (2,48–6,36)***	0,92
	14 oder jünger	REF	–
*Geschlecht*	Weiblich	5,60 (3,72–7,49)***	0,91
	Männlich	REF	
*Migrationshintergrund*	Zweiseitig	2,78 (0,48–5,08)*	1,11
	Einseitig	0,37 (−1,53–2,26)	0,92
	Keiner	REF	–
*Bildung* *Mutter*	Unbekannt	−0,05 (−2,80–2,70)	1,33
	Niedrig	1,99 (−2,19–6,16)	2,01
	Mittel	0,83 (−0,95–2,61)	0,86
	Hoch	REF	–
*Bildung* *Vater*	Unbekannt	−1,61 (−4,34–1,12)	1,32
	Niedrig	0,12 (−3,99–4,22)	1,98
	Mittel	−0,49 (−2,44–1,46)	0,94
	Hoch	REF	–
*Schulform*	Nichtgymnasial	14,88 (10,31–19,44)***	2,21
	Gymnasial	REF	
*Schulische* *Leistungen*	Niedrig	3,25 (1,62–4,87)***	0,78
	Mittel	1,89 (0,32–3,46)*	0,76
	Hoch	REF	–
*Rauchstatus*	Wöchentlich	2,40 (−1,01–5,82)	1,65
	Gelegentlich	2,21 (0,54–3,88)*	0,81
	Niemals	REF	–
*Elterliches* *Rauchen*	≥2 Raucher	2,00 (−1,04–5,04)	1,47
	1 Raucher	0,69 (−1,98–3,35)	1,29
	Ex-Raucher	−1,51 (−3,69–0,67)	1,06
	Keiner	REF	–
*Rauchende* *Freunde*	Einige	6,63 (4,15–9,10)***	1,20
	Die meisten	14,54 (11,00–18,08)***	1,71
	Alle	32,50 (22,45–42,55)***	4,86
	Keiner	REF	–

1,00 = Referenzkategorie (*REF*). ***-*p* < 0,001, **-*p* < 0,01, *‑*p* < 0,05

^a^Die durchschnittlich marginalen Effekte (AMEs) können in %‑Unterschieden interpretiert werden

^b^Für alle multivariaten Berechnungen wurden robuste Standardfehler (RSE) im Hinblick auf die Schulzugehörigkeit berechnet, um der geclusterten Datenstruktur gerecht zu werden

**Table 3:** 

	*N* = 2393	Modell 2: Schüler*innen	
		AMEs (KI95 %)^a^	RSE^b^
**Unabhängige Variable (Exposition)**			
Schulische Tabakkontrolle	(Skalenwert 0–6) Schüler*innen	−1,69 (−2,52–−0,86)*	0,40
**Kontroll- und Mediatorvariablen**			
*Erhebungsjahr*	2017	−11,58 (−15,54–−7,63)***	1,91
	2013	REF	
*Alter*	16 oder älter	7,58 (3,46–11,70)**	1,99
	15	4,08 (2,17–5,99)***	0,92
	14 oder jünger	REF	–
*Geschlecht*	Weiblich	5,41 (3,42–7,40)***	0,96
	Männlich	REF	
*Migrationshintergrund*	Zweiseitig	2,56 (0,38–4,74)*	1,05
	Einseitig	0,31 (−1,51–2,14)	0,88
	Keiner	REF	–
*Bildung* *Mutter*	Unbekannt	0,15 (−2,61–2,91)	1,33
	Niedrig	2,25 (−1,90–6,40)	2,00
	Mittel	1,34 (−0,45–3,13)	0,87
	Hoch	REF	–
*Bildung* *Vater*	Unbekannt	−1,43 (−4,15–1,30)	1,32
	Niedrig	0,28 (−3,85–4,42)	2,00
	Mittel	−0,18 (−2,11–1,77)	0,94
	Hoch	REF	–
*Schulform*	Nichtgymnasial	15,03 (10,13–19,93)***	2,37
	Gymnasial	REF	
*Schulische* *Leistungen*	Niedrig	3,43 (1,75–5,11)***	0,81
	Mittel	2,08 (0,52–3,64)*	0,75
	Hoch	REF	–
*Rauchstatus*	Wöchentlich	2,37 (−1,12–5,86)	1,69
	Gelegentlich	2,04 (0,32–3,76)*	0,83
	Niemals	REF	–
*Elterliches* *Rauchen*	≥2 Raucher	1,89 (−1,16–4,94)	1,48
	1 Raucher	0,57 (−2,21–3,35)	1,34
	Ex-Raucher	−1,59 (−3,76–0,58)	1,05
	Keiner	REF	–
*Rauchende* *Freunde*	Einige	6,58 (4,12–9,05)***	1,19
	Die meisten	14,13 (10,46–17,80)***	1,78
	Alle	31,46 (21,35–41,56)***	4,89
	Keiner	REF	–

1,00 = Referenzkategorie (*REF*). ***-*p* < 0,001, **-*p* < 0,01, *‑*p* < 0,05

^a^Die durchschnittlich marginalen Effekte (AMEs) können in %‑Unterschieden interpretiert werden

^b^Für alle multivariaten Berechnungen wurden robuste Standardfehler (RSE) im Hinblick auf die Schulzugehörigkeit berechnet, um der geclusterten Datenstruktur gerecht zu werden

Eine striktere schulische Tabakkontrolle ist in beiden Modellen (Tab. 2 und 3) konsistent mit einer geringeren schulischen Raucherprävalenz, sowohl für die von Lehrer- (AME: −3,54 KI95 % −6,49 bis −0,58) als auch Schüler*innen (AME: −1,69 KI95 % −2,52 bis −0,86) berichteten Tabakkontrolle assoziiert. Wenn der Skalenwert der Tabakkontrolle um eine Einheit steigt, reduziert sich die wahrgenommene Raucherprävalenz demzufolge um ca. 2–4 %. Ältere Heranwachsende (+7 %), Mädchen (+5 %), Jugendliche mit beidseitigem Migrationshintergrund (+3 %) und schwachen (+3 %) oder mittelmäßigen (+2 %) schulischen Leistungen sowie gelegentliche Raucher*innen (+2 %) korrelieren konsistent mit der Zielvariable dahin gehend, als dass sie eine signifikant höhere Tabakprävalenz für ihre Schulumwelt berichten (Tab. 2 und 3).

**Figure 1:**
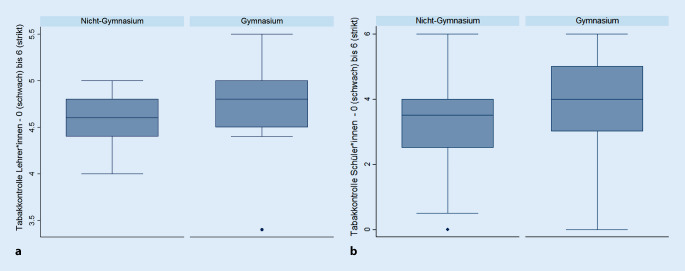

Die Anzahl rauchender Freunde (z. B. „die meisten“ mit +14 %: AME: 14,13 KI95 % 10,46–17,80) und der Schultyp sind bezüglich des Ausmaßes die relevantesten Determinanten für eine hoch eingeschätzte schulische Raucherprävalenz. Nichtgymnasiale Schulformen berichten eine 15 % (AME: 15,03 KI95 % 10,13–19,93) höhere Tabakprävalenz im Vergleich zu Gymnasien (Tab. 3), was wiederum mit der strikter implementierten Tabakkontrolle an Gymnasien (Abb. 1) korrespondiert.

## Diskussion

Die vorliegende Studie untersuchte die Assoziation zwischen schulischer Tabakkontrollpolitik und wahrgenommener Raucherprävalenz im Kontext einer deutschen Metropolregion.

### Zentrale Befunde

Die Ergebnisse deuten sowohl auf positive Entwicklungen als auch auf problematische soziale Ungleichheiten hin. Zum einem haben sich die wahrgenommene und die tatsächliche (selbstberichtete) Raucherprävalenz unter Jugendlichen zwischen beiden Erhebungswellen deutlich reduziert. In den adjustierten Modellen kann man eine durchschnittliche Reduzierung der Tabakprävalenz von mindestens 12 % beobachten. Zum anderen ist eine vonseiten der Lehrer- und Schülerschaft als schwach bewertete schulische Tabakkontrolle mit höherer schulischer Tabakprävalenz assoziiert. Die schulischen Durchsetzungspraktiken des Rauchverbots und die Schultypen tragen signifikant zu Ungleichheiten in der Prävalenz rauchender Jugendlicher bei. Es sind vor allem nichtgymnasiale Schulformen, welche mit etwa 15 % höherer geschätzter Raucherprävalenz exponiert sind.

### Interpretation

Der Mittelwert der durch die Heranwachsenden wahrgenommenen schulischen Raucherprävalenz liegt beim Wert von 30 %. Dies entspricht ungefähr dem Anteil der jugendlichen Jemals-Raucher*innen (33 %) unserer Hannoveraner Stichprobe sowie der durchschnittlichen Raucherprävalenz der Erwachsenenbevölkerung (ca. 28 %) Deutschlands [43]. Tabakprävalenzen im Jugendalter – im Sinne des regelmäßigen bzw. wöchentlichen Rauchens – liegen laut einer Auswertung des Robert Koch-Instituts (RKI) in 3 Jugendsurveys (BZgA, KiGGS, HBSC) seit dem Jahr 2010 unter 15 % und sind seit 2 Jahrzehnten stetig rückläufig, jedoch begleitet von Ungleichheiten nach Schulform [9, 18]. In der vorliegenden SILNE-R-Stichprobe aus Hannover und Umgebung liegt die durchschnittliche wöchentliche Raucherprävalenz sogar unter 10 % und ist zwischen beiden Erhebungszeitpunkten regressiv, wobei auch hier schulformspezifische Unterschiede deutlich werden. Studien des RKI berichten für Deutschland 24 % an täglichen oder gelegentlich Rauchenden im Erwachsenenalter mit signifikanten Unterschieden nach Geschlecht und Bildungsniveau [17]. Die Deutsche Befragung zum Rauchverhalten (DEBRA) berichtet ebenfalls Ungleichheiten nach SES und eine Prävalenz von 28 % bei Personen, die älter als 14 Jahre sind [43]. Angesichts dieser Lage im Erwachsenenalter und der bekannten gesundheitlichen Langzeitfolgewirkungen des Rauchens gilt es, dementsprechend im Jugendalter – trotz regressiver Trends in der Raucherprävalenz – weiterhin effektive primär- und sekundärpräventive Maßnahmen im Setting Schule zu ergreifen, um die Denormalisierung des Rauchens voranzutreiben.

Die identifizierten Korrelate der vorliegenden Studie suggerieren Ungleichheiten nach Schultyp sowie implementierter schulischer Tabakkontrolle und verweisen damit auf die Relevanz alltäglich normativer Durchsetzungspraktiken im Setting schulischer Tabakprävention [24, 44]. Auch andere Studien kommen zum Ergebnis, dass an Schulen mit strikter Regelauslegung und Tabakkontrolle signifikant weniger rauchende Jugendliche auf dem Schulgelände anzutreffen sind [16, 26]. Die partielle Denormalisierung des Rauchens im Jugendalter lässt sich mithin sehr deutlich durch den kontextuellen Umweltfaktor Schultyp [38, 39] und die dort implementierten präventiven Strategien erklären [26].

Der Untersuchungsort Hannover konnte in einer qualitativen Studie im Vergleich zu progressiveren irischen (Dublin) und finnischen (Tampere) EU-Kommunen ferner als „defensiv-saturierter“ Implementierungstyp herausgearbeitet werden [28]. Dies legt wiederum nahe, dass auch im Angesicht sinkender Tabakprävalenzen im Jugendalter noch ein gewisses Potenzial hinsichtlich einer weiteren Reduzierung des jugendlichen Rauchens im lokalen und schulischen Kontext besteht. Eine Möglichkeit zur Erhöhung der Effektivität der Durchsetzung des Rauchverbots würde in der Einbindung schulexterner Fachkräfte bestehen, da das interne Schulpersonal aufgrund begrenzter Zeitressourcen und anderer Prioritäten im Schulalltag dies schlichtweg nicht allumfänglich zu leisten vermag [32]. Qualitative Studien verweisen darauf, dass Lehrer*innen aus Gründen der Skepsis gegenüber der Effektivität von sanktionierenden Tabakkontrollmaßnahmen und eines priorisierenden Fokus auf die Persönlichkeitsentwicklung der Jugendlichen eher davon absehen, die schulische Tabakkontrolle strikt umzusetzen [33, 45]. Für die Einbindung schulexterner Fachkräfte könnten im deutschen Kontext Mittel auf Basis des Präventionsgesetzes über die Krankenkassen bereitgestellt werden. Darüber hinaus wäre es möglich, das Tabakthema fernab des Gesundheitsaspektes nicht nur im Fach Biologie, sondern auch im Hinblick auf nachhaltigkeitsorientierte Erwägungen (u. a. Umweltverschmutzung durch Zigarettenkippen, Kinderarbeit im Tabakanbau, Ausbeutung von Arbeiter*innen im globalen Süden) in Fächern wie Ethik oder Sozialkunde respektive die Rolle von Tabakwerbung im Fach Kunst und somit in alternativen didaktischen Formaten kritisch aufzuarbeiten [46].

Die Synthese aus den Studien von Galanti et al.[29] und Schreuders et al.[24, 31] zu der Implementierung rauchfreier Schulen schlägt letztlich Folgendes vor, um eine optimale Effektivität schulischer Tabakkontrolle zu realisieren:

- die Rauchverbote sollten möglichst widerspruchsfrei und einheitlich für alle anwesenden Personen in der Schule (im Gebäude, auf dem Gelände etc.) und möglichst auch in der schulnahen Umgebung gelten,
- die jeweiligen Maßnahmen sollten regelmäßig im Hinblick auf ihre Zwecke und gesundheitspolitische Legitimität vonseiten der Schulleitung kommuniziert werden,
- das Verantwortungsgefühl des Personals sollte geweckt werden und keinerlei Raum für nachgiebige Ausnahmen (z. B. für das Schulpersonal) gestattet werden,
- die Maßnahmen müssen konsistent umgesetzt und eher mit „progressiv ermutigenden“ statt „drakonischen“ disziplinären Maßnahmen sanktioniert werden,
- letztlich sollten sozial benachteiligte Risikogruppen (z. B. nichtgymnasiale Schulformen) unterstützt werden, die auch das größte Suchtpotenzial aufweisen und damit am ehesten gegen die schulischen Tabakkontrollmaßnahmen verstoßen [31].

Die elementare Hürde der Umsetzung schulischer Tabakkontrolle besteht primär für sozial benachteiligte Schulformen mit niedrigem SES [32], da aus der sozialepidemiologischen Forschung zu Tabak der hinderliche Einfluss eines geringen SES bekannt ist [7, 8, 16]. Jüngere Trenddaten für Deutschland zeigen zudem eine Persistenz von SES-Unterschieden in der häuslichen Passivrauchbelastung auf [13, 18]. Dies betrifft ebenso andere private Kontexte wie die toxische Tabakexposition im Automobil [36]. Es lassen sich in vielen Studien Ungleichheiten in der häuslichen Exposition und Permissivität gegenüber dem Rauchen für die elterliche Bildung belegen [12, 13, 18, 36], was auf die herausragende Relevanz schulischer Tabakkontrolle als präventives Gegengewicht in diesem Kontext und als Strategie zur Reduzierung gesundheitlicher Ungleichheiten verweist.

Die dokumentierten Ergebnisse zum Schultyp und zu rauchenden Freunden sind konsistent mit der sozialen Netzwerkforschung zum jugendlichen Rauchen [34, 47], da sie peergroup- und schulspezifische Unterschiede in der Exposition gegenüber einer permissiven Rauchumwelt aufzeigen [48]. Dies verweist auf die Relevanz freundschaftlicher Netzwerke für das jugendliche Rauchverhalten und deren Rauchkontexte [15, 16, 34, 40]. Der dokumentierte Geschlechter- und Altersunterschied im Hinblick auf die erhöht wahrgenommene Raucherprävalenz an der Schule könnte sich dadurch erklären lassen, dass Mädchen im Vergleich zu gleichaltrigen Jungen in höherem Maße mit älteren Teenagern und damit tendenziell auch mehr mit jugendlichen Raucher*innen im Schulalltag vernetzt sind [48].

### Limitationen

Die vorliegende wiederholte Querschnittsstudie unterliegt gewissen Beschränkungen hinsichtlich ihrer Aussagekraft, da sie nur Tendenzen und Zusammenhänge dokumentieren, jedoch keine Erklärungen über mögliche kausale Zusammenhänge aufzeigen kann. Die Studie mag an einigen Stellen in ihrer Interpretation eine Kausalität in der Wirkrichtung von schulischer Tabakkontrolle auf die schulische Tabakprävalenz implizieren. Es wäre im Sinne von umgekehrter Kausalität jedoch auch denkbar, dass Schüler- und Lehrerschaft eine schwache bzw. strikte Tabakkontrolle annehmen, wenn sie subjektiv viel respektive wenig Tabakkonsum beobachten. Die Stichprobe basiert auf selbstberichteten Angaben von 85 Angehörigen des Schulpersonals und im Durchschnitt 14-jährigen Jugendlichen einer westdeutschen Metropolregion, wodurch Prävalenzangaben und Befunde nicht für die nationale Ebene oder ältere Teenager generalisiert werden können. Aufgrund selbstberichteter Angaben ist nicht auszuschließen, dass die Angaben der befragten Jugendlichen zum eigenen Rauchen oder der wahrgenommenen Prävalenz durch verzerrende Effekte wie soziale Erwünschtheit unter- oder überschätzt worden sind [9, 18]. Die Rolle von Wasserpfeifen (Shisha), Cannabis oder elektronischen Alternativprodukten (z. B. E‑Zigarette, E‑Shisha) blieb in den Analysen unberücksichtigt. Das Rauchen von Shisha wird in der Gesundheitsgefährdung auch häufig dramatisch unterschätzt und erfährt gleichzeitig in zeitgenössischen Jugendkulturen – etwa über die Rap-Kultur vermittelt – eine zunehmende Popularität [49].

Durch den Verzicht auf multiple Imputation fallen ca. 30 % der Befragten aus der Bruttostichprobe weg (vgl. fehlende Werte Tab. 1), was zu Verzerrungen und einem Selektionsbias, etwa bei den Ergebnissen zum elterlichen SES und Rauchstatus, geführt haben kann. Einem relevanten Anteil der jugendlichen Befragten (ca. 24 %) ist der Bildungsabschluss von Mutter und Vater unbekannt gewesen, was zu verzerrten Ergebnissen beim SES-Indikator der Eltern geführt haben könnte. Die Ergebnisse nach Schultyp und damit nach Bildungsniveau der Schüler*innen zeigten jedoch plausible Resultate hinsichtlich von SES-Unterschieden. Die Rücklaufquote war an nichtgymnasialen Schulen aufgrund fehlender Einverständnisse der Eltern zudem lückenhafter als an Gymnasien oder Privatschulen, was die Verteilung von Prävalenzen und Tabakkontrollmaßnahmen beeinflussen kann [34]. Ferner ließe sich die nominale Erfassung der Schulformen kritisieren, jedoch haben auch Forschergruppen des RKI und der HBSC-Studie [9] darauf hingewiesen, dass aufgrund der gestiegenen Heterogenität der weiterführenden Schulformen eine zufriedenstellende (Aus‑)Differenzierung schwerfällt. Die Tabakkontrolle umfasst ferner nicht nur allein strikte Rauchverbote, sondern auch die Umsetzung von alternativen Aufklärungs- und Präventionsmaßnahmen (z. B. „Be smart – don’t start“) sowie das Vorhalten von Entwöhnungsangeboten. Diese Aspekte der schulischen Tabakkontrolle wurden in der vorliegenden quantitativen Studie – abgesehen von Inhalten im Schulcurriculum und Zugang zu Tabakprodukten in 100-Meter-Schulnähe – nicht systematisch erfasst, jedoch in anderen qualitativen Studien der Studiengruppe untersucht [28, 32].

## Fazit

Eine progressive schulische Tabakkontrollpolitik sollte den Fokus verstärkt auf sozial benachteiligte Risikogruppen, nichtgymnasiale Schulformen und jene Schulen legen, die Tabakkontrolle nicht strikt durchsetzen. Trotz messbar abnehmender Raucherprävalenz lassen sich persistente Ungleichheiten für nichtgymnasiale Schulformen und jene Schulen dokumentieren, die das Rauchverbot nicht strikt durchsetzen. Zukünftig bedarf es Forschung, die verändernde Umweltfaktoren (z. B. Popularisierung von E‑Zigaretten, Tabakerhitzern, E‑Shisha) im Jugend- und jüngeren Erwachsenenalter untersucht. Jene Produktinnovationen der transnationalen Tabakindustrie stellen ein ökonomisches Gegengewicht dar, das im Kontext der Denormalisierung von Tabak mögliche Renormalisierungen anderer Rauchprodukte auf (Jugend‑)Märkten vorantreibt [50].

## Supplementary Information




