# Supplementary material for: The association between school tobacco policies and the perceived smoking prevalence of adolescents
Source: Bundesgesundheitsblatt Gesundheitsforschung Gesundheitsschutz. 2020 Dec 7;64(1):91–101. [Article in German] doi: 10.1007/s00103-020-03261-1 (PMC7772164; doi:10.1007/s00103-020-03261-1)
Supplement: Supplementary file 1 [file 103_2020_3261_MOESM1_ESM.pdf]

FRAGEBOGEN

SILNER

Du, Deine Schule, Deine Gesundheit

MARTIN-LUTHER-UNIVERSITÄT
HALLE-WITTENBERG

LIEBE SCHÜLERIN, LIEBER SCHÜLER!

Über die heutige Befragung bist Du schon informiert. Dieser Fragebogen ist Teil einer internationalen Studie über das Rauchen bei europäischen Schüler/-innen, die von der Europäischen Kommission gefördert wird. Die Studie heißt SILNE-R. Die meisten Fragen handeln vom Rauchen, aber es gibt auch Fragen zu Deinen Freunden, Deiner Familie, dem Schulleben und Deiner Gesundheit.

Insgesamt wird der Fragebogen von ca. 10.000 Schüler/-innen in sieben verschiedenen Ländern beantwortet. Dieser Fragebogen wird für Deutschland vom Institut für Medizinische Soziologie (IMS) der Martin-Luther-Universität Halle-Wittenberg erhoben.

Vor Dir liegt jetzt der Fragebogen, in dem Du ankreuzen kannst, was Du machst und was Du über bestimmte Dinge denkst. Du hast zur Beantwortung der **ersten Seite** noch eine **Liste** aller Schüler in den Jahrgangsstufen 8 und 9 vor Dir liegen. Bitte schreibe in den Fragebogen **nur** die jeweilige ID der Schüler.

Die Teilnahme ist freiwillig. Es ist wichtig, dass Du so sorgfältig und offen wie möglich antwortest. Wenn Du den Fragebogen ausgefüllt hast, steck ihn bitte in den dafür vorgesehen Briefumschlag und klebe ihn zu. Die Schülerliste legst Du in den anderen Briefumschlag und klebst diesen ebenfalls zu.

Es handelt sich um einen **vertraulichen** Fragebogen, d.h. alle Informationen werden vertraulich behandelt. Auch Deine Lehrerinnen und Lehrer und Deine Eltern erfahren nicht, was Du im Fragebogen angibst.

Das ist kein Test! "Richtige" oder "falsche" Antworten gibt es nicht. Bitte lies Dir jede Frage sorgfältig durch und beantworte sie ehrlich und ohne lange nachzudenken. Wenn Du Schwierigkeiten hast, eine Antwort zu finden, wähle jene, die am ehesten für dich zutrifft.

Beispiel: Bist Du ein Junge oder ein Mädchen? Wenn Du ein Mädchen bist, mache bitte in dem rechten (2.) Kreis ein Kreuz.

1. ○ Junge 2. ○ Mädchen

Bei einigen Fragen musst Du auch etwas handschriftlich eintragen.

Wenn Du zustimmst an der Umfrage teilzunehmen,
dann mache bitte ein Kreuz in dem Kreis.

○ JA

Trage hier bitte Deine persönliche ID ein:

☐☐☐☐

VIELEN DANK, DASS DU AN UNSERER STUDIE TEILNIMMST!

1. **Bitte trage in das Kästchen Deine ID ein, die neben Deinem Namen auf der Schülerliste steht.**

1. ☐☐☐☐

DEINE MITSCHÜLER UND DEINE BESTEN FREUNDE

2. **Mit welchen Deiner Mitschüler (Jungen oder Mädchen) aus der 8. oder 9. Jahrgangsstufe arbeitest Du am liebsten zusammen oder fragst sie um einen Rat, z.B. bei Hausaufgaben?**

   Bitte suche die jeweiligen Namen aus der Schülerliste (max. 5) und trage nur die Schüler-ID in die Felder ein.

   Schüler-ID
   1. ☐☐☐☐
   2. ☐☐☐☐
   3. ☐☐☐☐
   4. ☐☐☐☐
   5. ☐☐☐☐

3. **Wer sind Deine besten Freunde in der 8. oder 9. Jahrgangsstufe?**

   Liste sie bitte unten auf. Jungen können Mädchen nennen, die ihre Freunde oder ihre Freundin sind; Mädchen können Jungen nennen, welche ihre Freunde oder ihr Freund sind (max. 5).

   Schüler-ID
   1. ☐☐☐☐
   2. ☐☐☐☐
   3. ☐☐☐☐
   4. ☐☐☐☐
   5. ☐☐☐☐

4. **Deine engsten Freunde hast Du…?**

   Kreuze nur eine Antwort an!

   1. ◯ fast nur in der Schule
   2. ◯ ungefähr genauso viele in der Schule wie außerhalb
   3. ◯ fast nur außerhalb der Schule
   4. ◯ ich habe keine engen Freunde

IN DEN FOLGENDEN FRAGEN GEHT ES UM DICH

5. **Wie alt bist Du?**

   Kreuze nur eine Antwort an!

   1. ◯ 12 Jahre 2. ◯ 13 Jahre 3. ◯ 14 Jahre 4. ◯ 15 Jahre
   5. ◯ 16 Jahre 6. ◯ 17 Jahre 7. ◯ 18 Jahre 8. ◯ 19 Jahre und älter

6. **Du bist…**

   Kreuze nur eine Antwort an!

   1. ◯ ...ein Mädchen 2. ◯ ...ein Junge

7. **In welchem Land bist Du geboren?**

   1. ◯ In Deutschland ▸ WEITER MIT FRAGE 9.
   2. ◯ In einem anderen Land: ____________________ ▸ WEITER MIT FRAGE 8.

8. **Wenn Du in einem anderen Land geboren bist, in welchem Jahr bist Du nach Deutschland gezogen?**

   Schreib die Jahreszahl in die Kästchen!

   Jahr ☐☐☐☐

GESUNDHEIT UND LEBENSSTIL

Die folgenden Fragen beschäftigen sich mit Deiner Gesundheit, Deinem Körper und Dingen, die Du tust.

9. Würdest Du sagen, Deine Gesundheit ist…? *Kreuze nur eine Antwort an!*

1. ◯ ausgezeichnet
2. ◯ gut
3. ◯ einigermaßen
4. ◯ schlecht

10. Wenn Du an die letzte Woche denkst…

Kreuze nur eine Antwort in jeder Zeile an!

	nie (I)	selten (II)	manchmal (III)	oft (IV)	immer (V)
1. Bist Du voller Energie gewesen?	◯	◯	◯	◯	◯
2. Hast Du dich traurig gefühlt?	◯	◯	◯	◯	◯
3. Hast Du dich einsam gefühlt?	◯	◯	◯	◯	◯
4. Hast Du genug Zeit für dich selbst gehabt?	◯	◯	◯	◯	◯
5. Konntest Du in deiner Freizeit die Dinge machen, die du tun wolltest?	◯	◯	◯	◯	◯
6. Haben Deine Mutter/dein Vater dich gerecht behandelt?	◯	◯	◯	◯	◯
7. Hast Du mit deinen Freunden Spaß gehabt?	◯	◯	◯	◯	◯
8. Konntest Du gut aufpassen?	◯	◯	◯	◯	◯

	überhaupt nicht (I)	ein wenig (II)	mittelmäßig (III)	ziemlich (IV)	sehr (V)
9. Hast Du dich fit und wohl gefühlt?	◯	◯	◯	◯	◯
10. Bist Du in der Schule gut zurechtgekommen?	◯	◯	◯	◯	◯

SILNER

11. Im Folgenden geht es um Deine körperliche Aktivität.

Während einer typischen Woche:
Wie viele Stunden bist Du an den jeweiligen Tagen im Durchschnitt körperlich aktiv?

Gemeint ist damit körperliche Aktivität beim Schulsport, mittags, am Abend und in der Freizeit. Starke körperliche Aktivitäten können Joggen, Radfahren, Individual- und Mannschaftssport (Schwimmen, Fußball, Tennis, Basketball, Handball, etc.), schnelles Tanzen und sonstige körperliche Betätigungen sein, die Deinen Pulsschlag erhöhen, Dich schwer atmen lassen oder zum Schwitzen bringen.

Kreuze nur eine Antwort in jeder Zeile an!

	keine (I)	30 Minuten (II)	1 Stunde (III)	1 ½ Stunden (IV)	2 Stunden (V)	2 ½ Stunden (VI)	3 Stunden oder mehr (VII)
1. Montag	◯	◯	◯	◯	◯	◯	◯
2. Dienstag	◯	◯	◯	◯	◯	◯	◯
3. Mittwoch	◯	◯	◯	◯	◯	◯	◯
4. Donnerstag	◯	◯	◯	◯	◯	◯	◯
5. Freitag	◯	◯	◯	◯	◯	◯	◯
6. Samstag	◯	◯	◯	◯	◯	◯	◯
7. Sonntag	◯	◯	◯	◯	◯	◯	◯

12. Bitte denke an die letzten 12 Monate zurück: Wie oft hast Du Alkohol getrunken (mehr als einen Schluck)?

"Ein alkoholisches Getränk" bedeutet: 1 normal große Flasche, Dose oder Glas Bier, 1 Glas Wein, 1 Spirituose "Shot" (Rum, Whisky, etc.) oder ein Mixgetränk (1 Spirituose mit Saft, Energydrink, etc.).

Kreuze nur eine Antwort an!

1. ◯ Ich habe in den letzten 12 Monaten keinen Alkohol getrunken.
2. ◯ Weniger als einmal im Monat.
3. ◯ Einmal im Monat.
4. ◯ 2- bis 3-mal im Monat.
5. ◯ Einmal in der Woche.
6. ◯ Mehrmals in der Woche.
7. ◯ Täglich.

13. Wie oft hast Du in den letzten 12 Monaten bei einer Gelegenheit 5 oder mehr alkoholische Getränke getrunken?

Kreuze nur eine Antwort an!

1. ◯ Ich hatte keinen Alkohol oder habe weniger als 5 alkoholische Getränke getrunken.
2. ◯ Weniger als einmal im Monat.
3. ◯ Einmal im Monat.
4. ◯ 2- bis 3-mal im Monat.
5. ◯ Einmal in der Woche.
6. ◯ 2-mal oder mehrmals in der Woche.

14. Wie oft warst Du in den letzten 12 Monaten in Bars, Clubs oder Diskotheken?

Kreuze nur eine Antwort an!

1. ◯ Nie
2. ◯ Weniger als einmal im Monat.
3. ◯ 2- bis 3-mal im Monat.
4. ◯ Einmal in der Woche.
5. ◯ 2-mal oder mehrmals in der Woche.

15. Wenn Du an die letzten 12 Monate denkst, wie oft hast Du Marihuana oder Haschisch konsumiert?

Kreuze nur eine Antwort an!

1. ◯ Ich habe noch nie Marihuana/Haschisch genommen.
2. ◯ Ich habe schon mal Marihuana/Haschisch genommen, aber nicht in den letzten 12 Monaten.
3. ◯ Weniger als einmal im Monat.
4. ◯ Einmal im Monat.
5. ◯ 2- bis 3-mal im Monat.
6. ◯ Einmal in der Woche.
7. ◯ Mehrmals in der Woche.
8. ◯ Täglich

DEINE ERFAHRUNGEN MIT DEM RAUCHEN

Die folgenden Fragen beschäftigen sich mit dem Rauchen (sowohl Zigaretten, Selbstdrehen von Zigaretten, Zigarren und Zigarillos als auch kleine Zigarren). Diese Fragen betreffen nicht E-Zigaretten, dazu werden wir später fragen. Es ist für uns wichtig, mehr über die Erfahrungen und Einstellungen zum Rauchen zu erfahren.

16. Hast Du jemals das Rauchen ausprobiert, wenn auch nur ein paar Züge?

Kreuze nur eine Antwort an!

1. ◯ Nein ► WEITER MIT FRAGE **28.**
2. ◯ Ja

17. Wie alt warst Du, als Du das erste Mal geraucht hast, wenn auch nur ein paar Züge?

Kreuze nur eine Antwort an!

1. ◯ Ich habe noch nie geraucht
2. ◯ 9 Jahre oder jünger
3. ◯ 10 Jahre
4. ◯ 11 Jahre
5. ◯ 12 Jahre
6. ◯ 13 Jahre
7. ◯ 14 Jahre
8. ◯ 15 Jahre
9. ◯ 16 Jahre
10. ◯ 17 Jahre
11. ◯ 18 Jahre und älter

18. Wie viele Zigaretten hast Du in den letzten 30 Tagen geraucht?

Kreuze nur eine Antwort an!

1. ◯ Ich habe noch nie geraucht.
2. ◯ gar keine
3. ◯ 1 bis 2 Zigaretten während der letzten 30 Tage
4. ◯ 1 bis 2 Zigaretten pro Woche
5. ◯ 3 bis 7 Zigaretten pro Woche
6. ◯ 1 bis 5 Zigaretten pro Tag
7. ◯ 6 bis 10 Zigaretten pro Tag (ungefähr eine halbe Schachtel)
8. ◯ 11 bis 20 Zigaretten pro Tag (ungefähr eine Schachtel)
9. ◯ 21 bis 30 Zigaretten pro Tag
10. ◯ mehr als 30 Zigaretten pro Tag

19. Wenn Du an die letzten 12 Monate denkst: Hat sich Dein Rauchverhalten verändert?

Kreuze nur eine Antwort an!

1. ◯ Ja, ich habe mit dem Rauchen angefangen.
2. ◯ Ja, ich rauche mehr als letztes Jahr.
3. ◯ Ja, ich rauche weniger als letztes Jahr.
4. ◯ Nein, ich rauche genauso viel wie letztes Jahr.
5. ◯ Nein, ich habe nie geraucht.

20. Wissen Deine Eltern, dass Du rauchst oder geraucht hast?

Kreuze nur eine Antwort an!

1. ◯ Ja.
2. ◯ Nein.
3. ◯ Weiß ich nicht.
4. ◯ Nein, ich habe nie geraucht.

21. Hast Du jemals versucht, mit dem Rauchen aufzuhören?

Kreuze nur eine Antwort an!

1. ◯ Ich habe noch nie geraucht.
2. ◯ Ich habe nur ein paar Mal geraucht.
3. ◯ Ich habe nie versucht aufzuhören.
4. ◯ Ich habe mindestens einmal versucht aufzuhören.
5. ◯ Ich habe bereits aufgehört zu rauchen. ► WEITER MIT FRAGE 28.

22. An wie vielen Tagen hast Du in den letzten 30 Tagen geraucht, wenn auch nur ein paar Züge?

Kreuze nur eine Antwort an!

1. ◯ gar keine ► WEITER MIT FRAGE 28.
2. ◯ 1 oder 2 Tage
3. ◯ 3 bis 5 Tage
4. ◯ 6 bis 10 Tage
5. ◯ 11 bis 20 Tage
6. ◯ (fast) täglich

SILNER

23. Wo rauchst Du normalerweise?

Kreuze alle Antworten an, die zutreffen!

1. ◯ zu Hause
2. ◯ auf dem Schulgelände
3. ◯ nur außerhalb der Schule
4. ◯ bei der Arbeit
5. ◯ bei Freunden zu Hause
6. ◯ Café, Bar, Kneipe, Nachtclub
7. ◯ Einkaufszentrum
8. ◯ an anderen öffentlichen Orten (z.B. Parks, Straßen)

24. Wie oft rauchst Du in der Schule bzw. auf dem Schulgelände?

Kreuze nur eine Antwort an!

1. ◯ täglich
2. ◯ Die meisten Tage der Woche.
3. ◯ Einige Tage in der Woche.
4. ◯ nie

25. Woher hast du deine Zigaretten in den letzten 30 Tagen normalerweise bekommen?

Kreuze alle Antworten an, die zutreffen!

1. ◯ Ich habe in den letzten 30 Tagen keine Zigaretten geraucht.
2. ◯ Ich habe sie in einem Geschäft/Kiosk gekauft.
3. ◯ Ich habe sie an einem Zigarettenautomaten gekauft.
4. ◯ Ich habe sie an einer Tankstelle gekauft.
5. ◯ Ich habe sie von meinen Eltern oder Geschwistern.
6. ◯ Ich habe sie von Freunden bekommen.
7. ◯ Ich habe sie von Freunden gekauft.
8. ◯ Ich habe sie von meinen Eltern oder Geschwistern gekauft.
9. ◯ Ich habe sie im Internet gekauft.
10. ◯ Ich habe andere Minderjährige (jünger als 18 Jahre) gefragt, ob sie welche für mich kaufen.
11. ◯ Ich habe eine mir bekannte erwachsene Person gefragt, ob sie welche für mich kauft.
12. ◯ Ich habe eine mir unbekannte erwachsene Person gefragt, ob sie welche für mich kauft.
13. ◯ Ich habe sie auf einem anderen Weg bekommen.

26. Hast Du in den letzten 30 Tagen Zigaretten in einem Geschäft, von einem Straßenverkäufer, einem Zigarettenautomaten, einer Tankstelle oder im Internet gekauft oder versucht zu kaufen?

Kreuze nur eine Antwort an!

1. ◯ Ja, ich habe Zigaretten gekauft.
2. ◯ Ja, ich habe es versucht, aber es hat nicht geklappt.
3. ◯ Nein, ich habe es nicht versucht.
4. ◯ Nein, das habe ich noch nie versucht.

27. Was denkst Du: Findest Du es schwierig Zigaretten in einem Geschäft, von einem Straßenverkäufer, einem Zigarettenautomaten, einer Tankstelle oder im Internet zu kaufen?

Kreuze nur eine Antwort an!

1. ◯ Es ist sehr schwierig.
2. ◯ Es ist ziemlich schwierig.
3. ◯ Es ist ziemlich einfach.
4. ◯ Es ist sehr einfach.
5. ◯ Ich kaufe keine Zigaretten.

SILNE-R

ALLE SCHÜLER BEANTWORTEN DIE FOLGENDEN FRAGEN

28. Denkst Du, dass Du bald eine Zigarette rauchen wirst?

Kreuze nur eine Antwort an!

1. ◯ bestimmt nicht
2. ◯ wahrscheinlich nicht
3. ◯ wahrscheinlich ja
4. ◯ ja, auf jeden Fall

29. Wie schwierig wäre es an Zigaretten zu kommen, wenn Du es möchtest?

Kreuze nur eine Antwort an!

1. ◯ sehr schwierig
2. ◯ ziemlich schwierig
3. ◯ ziemlich einfach
4. ◯ sehr einfach

30. Wenn einer Deiner Freunde Dir eine Zigarette anbieten würde, würdest Du sie rauchen?

Kreuze nur eine Antwort an!

1. ◯ bestimmt nicht
2. ◯ wahrscheinlich nicht
3. ◯ wahrscheinlich ja
4. ◯ ja, auf jeden Fall

31. Wie viele Deiner besten und engsten Freunde rauchen?

Kreuze nur eine Antwort an!

1. ◯ keiner davon
2. ◯ einige davon
3. ◯ die meisten davon
4. ◯ alle davon

32a. Wie oft siehst Du Schüler auf dem Schulgelände rauchen?

Kreuze nur eine Antwort an!

1. ◯ nie
2. ◯ manchmal
3. ◯ oft
4. ◯ immer

32b. Wie oft siehst Du Lehrer auf dem Schulgelände rauchen?

Kreuze nur eine Antwort an!

1. ◯ nie
2. ◯ manchmal
3. ◯ oft
4. ◯ immer

33. Wie oft siehst Du Leute direkt vor der Schule rauchen?

Kreuze nur eine Antwort an!

1. ◯ nie
2. ◯ manchmal
3. ◯ oft
4. ◯ immer

34. Hast Du in den letzten 6 Monaten Leute an diesen Orten rauchen gesehen?

Kreuze nur eine Antwort in jeder Zeile an!

	Ja (I)	Nein (II)	Ich halte mich dort nie auf (III)
1. zu Hause	◯	◯	◯
2. bei Freunden zu Hause	◯	◯	◯
3. im Restaurant, Fast Food-Laden (Döner, etc.)	◯	◯	◯
4. Bars, Kneipen, Cafés, Clubs, Diskotheken	◯	◯	◯
5. auf Bahnhöfen oder an Bushaltestellen	◯	◯	◯
6. in Freizeit- oder Sporteinrichtungen	◯	◯	◯

SILNER

35. Was schätzt Du? Wie viele Leute in Deinem Alter rauchen in etwa in Deiner Stadt?

Kreuze nur eine Antwort an!

0%	5%	10%	20%	30%	40%	50%	60%	70%	80%	90%	100%
◯	◯	◯	◯	◯	◯	◯	◯	◯	◯	◯	◯
1.	2.	3.	4.	5.	6.	7.	8.	9.	10.	11.	12.

36. Was schätzt Du? Wie viele Schüler in Deinem Alter rauchen in etwa an Deiner Schule?

Kreuze nur eine Antwort an!

0%	5%	10%	20%	30%	40%	50%	60%	70%	80%	90%	100%
◯	◯	◯	◯	◯	◯	◯	◯	◯	◯	◯	◯
1.	2.	3.	4.	5.	6.	7.	8.	9.	10.	11.	12.

37. Wie würden diese Leute reagieren, wenn sie wüssten, dass Du rauchst?

Kreuze nur eine Antwort in jeder Zeile an!

	sie würden es akzeptieren (I)	sie würden es nicht akzeptieren (II)	sie würden es eher ablehnen (III)	sie würden es strikt ablehnen (IV)
1. Deine besten Freunde	◯	◯	◯	◯
2. Deine Eltern	◯	◯	◯	◯
3. Deine Lehrer	◯	◯	◯	◯

38. Was meinst Du dazu? Rauchen erhöht Deine Chancen …

Kreuze nur eine Antwort in jeder Zeile an!

	stimme voll und ganz zu (I)	stimme eher zu (II)	stimme nicht zu (III)	stimme überhaupt nicht zu (IV)
1. cool auszusehen.	○	○	○	○
2. Probleme zu bekommen.	○	○	○	○
3. sich entspannt zu fühlen.	○	○	○	○
4. beliebt zu sein.	○	○	○	○
5. erwachsen auszusehen.	○	○	○	○
6. Lungenkrebs zu bekommen.	○	○	○	○
7. männlich auszusehen.	○	○	○	○
8. weiblich auszusehen.	○	○	○	○
9. Gewicht abzunehmen und dünn zu bleiben.	○	○	○	○
10. Probleme beim Atmen zu bekommen.	○	○	○	○
11. Falten zu bekommen.	○	○	○	○
12. sexy und attraktiv auszusehen.	○	○	○	○
13. einen festen Freund oder feste Freundin zu bekommen.	○	○	○	○
14. schlechter Sport zu treiben.	○	○	○	○

39. Bitte gebe an, was Leute über Raucher und das Rauchen denken.

Kreuze nur eine Antwort in jeder Zeile an!

	stimme voll und ganz zu (I)	stimme eher zu (II)	stimme nicht zu (III)	stimme überhaupt nicht zu (IV)
1. Die meisten Leute würden keinen Babysitter anstellen, der Raucher ist.	○	○	○	○
2. Die meisten Nichtraucher würden nur ungern mit Raucher ausgehen.	○	○	○	○
3. Die meisten Leute denken, dass Rauchen etwas für Verlierer ist.	○	○	○	○
4. Die meisten Leute denken schlecht über Raucher.	○	○	○	○

40. An wie vielen Tagen bist Du in der letzten Woche in einem Auto mitgefahren, in dem jemand geraucht hat?

Kreuze nur eine Antwort an!

1. ○ gar keinen
2. ○ 1 oder 2 Tagen
3. ○ 3 oder 4 Tagen
4. ○ 5 oder 6 Tagen
5. ○ An allen 7 Tagen
6. ○ Ich bin in der letzten Woche in keinem Auto mitgefahren.
7. ○ weiß ich nicht

DIE FOLGENDEN FRAGEN BESCHÄFTIGEN SICH MIT E-ZIGARETTEN.

41. Welche der folgenden Aussagen über E-Zigaretten trifft auf Dich zu?

Kreuze nur eine Antwort an!

1. ◯ Ich habe nie eine E-Zigarette probiert. ➤WEITER MIT FRAGE 44.
2. ◯ Ich habe eine E-Zigarette 1- bis 2-mal probiert.
3. ◯ Ich habe mehrfach eine E-Zigarette probiert.
4. ◯ Ich rauche eine E-Zigarette mindestens einmal im Monat.
5. ◯ Ich rauche eine E-Zigarette mindestens einmal in der Woche.
6. ◯ Ich rauche eine E-Zigarette täglich.
7. ◯ Ich weiß nicht, was E-Zigaretten sind. ➤WEITER MIT FRAGE 44.

42. Wenn Du schon einmal eine E-Zigarette probiert hast, aus welcher Substanz besteht sie?

Kreuze alle Antworten an, die zutreffen!

1. ◯ Flüssigkeiten mit Nikotin.
2. ◯ Flüssigkeiten ohne Nikotin.
3. ◯ Weiß ich nicht.
4. ◯ Ich habe E-Zigaretten nie probiert.

43. Als Du das erste Mal eine E-Zigarette geraucht hast, hast Du da schon normale Zigaretten geraucht?

Kreuze nur eine Antwort an!

1. ◯ Ich habe nie normale Zigaretten geraucht.
2. ◯ Ich habe Tabak probiert, aber nicht regelmäßig konsumiert.
3. ◯ Ich habe Tabak gelegentlich geraucht.
4. ◯ Ich habe Tabak regelmäßig geraucht.
5. ◯ Ich habe E-Zigaretten nie probiert.

DU UND DEINE SCHULE

Die folgenden Fragen beschäftigen sich mit dem Rauchen an Deiner Schule.

44. Was meinst Du: Was denken die Lehrer über die rauchenden Jugendlichen an Deiner Schule?

Kreuze nur eine Antwort an!

1. ◯ Die meisten Lehrer akzeptieren es.
2. ◯ Die meisten Lehrer achten nicht darauf.
3. ◯ Die meisten Lehrer lehnen es eher ab.
4. ◯ Die meisten Lehrer lehnen es strikt ab.
5. ◯ Weiß ich nicht.

45. Dürfen Schüler auf dem Schulgelände rauchen?

Kreuze nur eine Antwort an!

1. ◯ Nein, Schüler dürfen nicht rauchen. Diese Regel wird strikt durchgesetzt.
2. ◯ Nein, Schüler dürfen nicht rauchen, aber diese Regel wird nicht strikt durchgesetzt.
3. ◯ Ja, Schüler dürfen in bestimmten Bereichen rauchen.
4. ◯ Ja, Schüler dürfen überall auf dem Schulgelände rauchen.
5. ◯ Weiß ich nicht.

46. Dürfen Lehrer auf dem Schulgelände rauchen?

Kreuze nur eine Antwort an!

1. ◯ Nein, Lehrer dürfen nicht rauchen.
2. ◯ Ja, Lehrer dürfen in bestimmten Bereichen rauchen.
3. ◯ Ja, Lehrer dürfen überall auf dem Schulgelände rauchen.
4. ◯ Weiß ich nicht.

47. Was würdest Du erwarten: Was sollte mit Schülern passieren, die sich in den letzten sechs Monaten nicht an die Rauchregeln an Deiner Schule gehalten haben?

Kreuze alle Antworten an, die zutreffen!

1. ◯ Schriftliche oder mündliche Verwarnung.
2. ◯ Belehrendes Gespräch.
3. ◯ Eltern informieren.
4. ◯ Vorladung beim Schuldirektor.
5. ◯ Verpflichtung zu freiwilliger Arbeit in der Schule.
6. ◯ Nachsitzen/Unterrichtsausschluss.
7. ◯ Unterrichtsauschluss.
8. ◯ Nichts.
9. ◯ Weiß ich nicht.
10. ◯ sonstiges (bitte nennen): ____________________

DEINE SCHULERFAHRUNG

Die folgenden Fragen beschäftigen sich mit Dir und Deiner Schule.

48. Seit wann gehst Du auf diese Schule?

Kreuze nur eine Antwort an!

1. ◯ Seit diesem Schuljahr (2016/2017).
2. ◯ Seit letztem Schuljahr (2015/2016).
3. ◯ Seit zwei Schuljahren (2014/2015).
4. ◯ Seit drei Schuljahren (2013/2014).
5. ◯ Früher.

SILNER

49. Welche der folgenden Antwortmöglichkeiten beschreiben Deine Noten während des letzten Schuljahres am besten?

Kreuze nur eine Antwort an!

1. ◯ überwiegend die Note 1
2. ◯ überwiegend die Note 2
3. ◯ überwiegend die Note 3
4. ◯ überwiegend die Note 4
5. ◯ überwiegend schlechter als die Note 4

50. Bitte kreuze an, inwieweit Du mit den Aussagen übereinstimmst oder nicht übereinstimmst.

Kreuze nur eine Antwort in jeder Zeile an!

	stimmt genau (I)	stimmt eher (II)	stimmt eher nicht (III)	stimmt überhaupt nicht (IV)
1. Ich fühle mich mit den Menschen an meiner Schule verbunden.	◯	◯	◯	◯
2. Ich fühle mich als ein Teil dieser Schule.	◯	◯	◯	◯
3. Ich bin glücklich, auf diese Schule zu gehen.	◯	◯	◯	◯
4. Ich finde unsere Lehrer behandeln uns gerecht.	◯	◯	◯	◯
5. Ich fühle mich an meiner Schule sicher.	◯	◯	◯	◯

DEINE FAMILIE

Die folgenden Fragen beschäftigen sich mit Deiner Familie und Deinem Zuhause.

51. In welchem Land ist Deine Mutter geboren?

1. ◯ in Deutschland
2. ◯ In einem anderen Land: ____________________

52. In welchem Land ist Dein Vater geboren?

1. ◯ in Deutschland
2. ◯ In einem anderen Land: ____________________

53. Welchen höchsten Schul- oder Bildungsabschluss hat Deine Mutter?

Kreuze nur eine Antwort an!

1. ◯ Hauptschulabschluss/Volksschulabschluss
2. ◯ Realschulabschluss
3. ◯ Abschluss Polytechnische Oberschule (POS, 10. Klasse)
4. ◯ Fachhochschulreife (Abschluss einer Fachoberschule)
5. ◯ Abitur (Gymnasium bzw. EOS)
6. ◯ Fachhochschule, Ingenieurschule
7. ◯ Universität, Hochschule
8. ◯ anderer Schulabschluss
9. ◯ Schule beendet ohne Schulabschluss
10. ◯ (noch) keinen Schulabschluss
11. ◯ Weiß ich nicht.

54. Welchen höchsten Schul- oder Bildungsabschluss hat Dein Vater?

Kreuze nur eine Antwort an!

1. ◯ Hauptschulabschluss/Volksschulabschluss
2. ◯ Realschulabschluss
3. ◯ Abschluss Polytechnische Oberschule (POS, 10. Klasse)
4. ◯ Fachhochschulreife (Abschluss einer Fachoberschule)
5. ◯ Abitur (Gymnasium bzw. EOS)
6. ◯ Fachhochschule, Ingenieurschule
7. ◯ Universität, Hochschule
8. ◯ anderer Schulabschluss
9. ◯ Schule beendet ohne Schulabschluss
10. ◯ (noch) keinen Schulabschluss
11. ◯ Weiß ich nicht.

SILNER

DEINE FAMILIE UND DEIN ZUHAUSE

Die nächsten Fragen beschäftigen sich mit Deiner Familie. Es gibt unterschiedliche Familien (zum Beispiel lebt nicht jeder mit beiden Eltern zusammen, manche leben nur mit einem Elternteil, manche sind an zwei Orten zu Hause oder leben in zwei verschiedenen Familien). Wir möchten gerne wissen, wie die Situation bei Dir ist.

Bitte beantworte die folgenden Fragen für Dein Zuhause, wo Du immer oder die meiste Zeit wohnst.

55. Welche der folgenden Personen leben bei Dir zu Hause?

Kreuze alle Antworten an, die zutreffen!

1. ◯ mein Vater
2. ◯ mein Stiefvater
3. ◯ meine Mutter
4. ◯ meine Stiefmutter
5. ◯ mein/meine (Stief-)Bruder/Brüder
6. ◯ meine (Stief-)Schwester/n
7. ◯ meine Großeltern
8. ◯ andere Verwandte
9. ◯ keine Verwandte
10. ◯ ich lebe allein
11. ◯ ich lebe im Internat

56. Welche Sprache sprichst Du am häufigsten zu Hause?

Kreuze nur eine Antwort an!

1. ◯ Deutsch
2. ◯ Eine andere Sprache: ____________________

REGELN ZUM RAUCHEN BEI DIR ZU HAUSE

Die folgenden Fragen beschäftigen sich mit Regeln zum Rauchen bei Dir zu Hause (der Ort, an dem Du die ganze oder die meiste Zeit wohnst).

57. Raucht jemand von den Personen, mit denen Du zusammen wohnst?

Kreuze nur eine Antwort in jeder Zeile an!

	Ja (I)	Nein (II)	Hat mit Rauchen aufgehört (III)	Trifft nicht zu (IV)
1. Vater	○	○	○	○
2. Stiefvater	○	○	○	○
3. Mutter	○	○	○	○
4. Stiefmutter	○	○	○	○
5. (Stief-) Bruder/Brüder	○	○	○	○
6. (Stief-) Schwester(n)	○	○	○	○
7. Großmutter	○	○	○	○
8. Großvater	○	○	○	○
9. Andere Verwandte	○	○	○	○
10. Keine Verwandte	○	○	○	○

58. Ist bei Dir zu Hause das Rauchen erlaubt??

Kreuze nur eine Antwort an!

1. ○ Niemand darf bei mir zu Hause rauchen.
2. ○ Das Rauchen bei mir zu Hause ist nur in bestimmten Bereichen erlaubt.
3. ○ Das Rauchen ist überall bei mir zu Hause erlaubt.
4. ○ Das weiß ich nicht.

59. Wie viel Geld hast Du normalerweise durch Taschengeld oder Jobs, wie z.B. Babysitten oder Zeitungen austragen, pro Woche zum Ausgeben oder Sparen zur Verfügung?

Gib den Betrag (z.B. 20€) bitte in Zahlen an!

.... **Euros**

60. Stell Dir vor, dass diese Leiter darstellt, wie die deutsche Gesellschaft aufgebaut ist.

- **Oben** auf der Leiter sind die Menschen, die am wohlhabendsten sind – sie haben das meiste Geld, die beste Ausbildung und eine Arbeitsstelle, die ihnen den größten Respekt einbringt.
- **Unten** auf der Leiter sind Menschen, die am wenigsten wohlhabend sind – sie haben eine geringe oder keine Ausbildung, keine Arbeit oder eine schlechtbezahlte Arbeitsstelle.

Nun stell Dir **Deine Familie** vor. Bitte teile uns mit, wo du glaubst, dass sich deine Familie auf dieser Leiter befindet.

Kreuze das Kästchen an, welches darstellt, wo sich Deine Familie auf dieser Leiter befinden würde.

10. ○ .... **am wohlhabendsten**
9. ○ ....
8. ○ ....
7. ○ ....
6. ○ ....
5. ○ ....
4. ○ ....
3. ○ ....
2. ○ ....
1. ○ ....
0. ○ .... **am wenigsten wohlhabend**

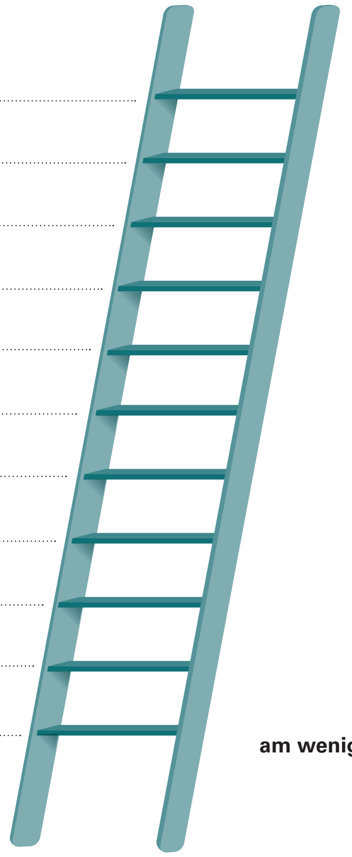

SILNER

61. Ich bin ziemlich sicher, dass ich …

Bitte machen Sie in jeder Zeile ein Kreuz!

	stimme überhaupt nicht zu (I)	stimme nicht zu (II)	stimme eher zu (III)	stimme voll und ganz zu (IV)
1. …gute Informationen über Gesundheit habe.	○	○	○	○
2. …leicht gesundheitsbezogene Informationen finde, die ich verstehen kann.	○	○	○	○
3. …ermessen kann, inwiefern meine eigenen Handlungen meine Umwelt beeinflussen.	○	○	○	○
4. …leicht Beispiele anführen kann, die gesundheitsfördernd sind.	○	○	○	○
5. …den Anweisungen von Personal aus dem Gesundheitswesen (z.B. Krankenschwester, Arzt, etc.) folgen kann.	○	○	○	○
6. …Begründungen anführen kann, die mein Gesundheitsverhalten erklären.	○	○	○	○
7. …wenn es notwendig erscheint, gesundheitsfördernde Inhalte meiner unmittelbaren Umgebung (Familien, Freunden, Leuten aus dem Ort, etc.) vermitteln kann.	○	○	○	○
8. …für gewöhnlich rausfinden kann, welche gesundheitsbezogenen Informationen richtig oder falsch sind.	○	○	○	○
9. …ermessen kann, inwiefern meine Verhaltensweisen meine Gesundheit beeinflussen.	○	○	○	○
10. …gesundheitsbezogene Informationen aus unterschiedlichen Quellen vergleichen kann.	○	○	○	○

Vielen Dank für Deine Teilnahme an unserer Studie.

Möchtest Du uns noch etwas mitteilen?

Dann hast Du hier Platz dafür:

....

....

....

....

....

....

....

....

....

....

....

MARTIN-LUTHER-UNIVERSITÄT
HALLE-WITTENBERG

SILNER

FRAGEBOGEN

SILNER

Du, Deine Schule, Deine Gesundheit

MARTIN-LUTHER-UNIVERSITÄT
HALLE-WITTENBERG

Enhancing the Effectiveness of Programs and Strategies to Prevent Smoking by Adolescents: A Realist Evaluation Comparing Seven European Countries (SILNE-R)

Studienverantwortlicher/beteiligte Wissenschaftler:

Prof. Matthias Richter (Martin-Luther-Universität Halle-Wittenberg)
Prof. Anton Kunst (Academic Medical Centre Amsterdam)
Prof. Vincnent Lorant (Université Catholique de Louvain)

Website: http://silne-r.ensp.org/

Zustand der Schule

Die folgenden Fragen beschäftigen sich mit Tabakinfrastruktur und dem sozialen Klima der Schule.

A. **Können Zigaretten oder Tabakwaren in weniger als 100m Entfernung von Ihrer Schule gekauft werden?**

1 ❑ nein

2 ❑ ja

B. **Wie schätzen Sie die Atmosphäre/das soziale Klima an Ihrer Schule ein?**

1 ❑ sehr gut

2 ❑ gut

3 ❑ eher gut

4 ❑ eher schlecht

5 ❑ schlecht

6 ❑ sehr schlecht

Rauchen

Die folgenden Fragen beschäftigen sich mit der Schulpolitik in Bezug auf das Rauchen bei Schüler-, Mitarbeiter-, und Besucher/-innen. Mit Mitarbeiter/-innen meinen wir Schulleiter, Lehrkräfte, Schulsekretär/-innen, Verpflegungspersonal und sonstiges Personal. Mit Besucher/-innen meinen wir keine Schüler/-innen und Mitarbeiter/-innen, sondern alle sonstigen Personen, einschließlich Angehörige der Gemeinde und Handwerker.

C. **Gibt es an Ihrer Schule Regeln, die das Rauchen verbieten?**

1 ❑ nein

2 ❑ ja

D. **Untersagen diese Regeln explizit folgenden Personen zu folgenden Zeiten das Rauchen?**

Kreuzen Sie nur ja oder nein pro Personengruppe an!

		Schüler/-innen		Mitarbeiter/-innen		Besucher/-innen	
		ja	nein	ja	nein	ja	nein
1.	während des Schulunterrichts	1 ❑	2 ❑	3 ❑	4 ❑	5 ❑	6 ❑
2.	außerhalb des Schulunterrichts	1 ❑	2 ❑	3 ❑	4 ❑	5 ❑	6 ❑

E. Untersagen diese Regeln explizit folgenden Personen an folgenden Orten das Rauchen?

Kreuzen Sie nur ja oder nein pro Personengruppe an

!

		Schüler/-innen		Mitarbeiter/-innen		Besucher/-innen	
		ja	nein	ja	nein	ja	nein
1.	Im Schulgebäude?	1❑	2❑	3❑	4❑	5❑	6❑
2.	Auf dem Schulgelände, einschließlich Parkplätzen und Sportplätzen?	1❑	2❑	3❑	4❑	5❑	6❑
3.	In Schulbussen oder anderen Verkehrsmitteln die von den Schülern genutzt werden?	1❑	2❑	3❑	4❑	5❑	6❑
4.	Außerhalb des Schulgeländes, an Schulveranstaltungen?	1❑	2❑	3❑	4❑	5❑	6❑

F. Seit wann bestehen Regeln zum Rauchen an Ihrer Schule?

1❑	5 Jahre oder länger
2❑	3 oder 4 Jahre
3❑	1 oder 2 Jahre
4❑	weniger als 1 Jahr

G. Ist Tabakwerbung verboten…

	ja	nein
…im Schulgebäude?	1❑	2❑
…auf dem gesamten Schulgelände, einschließlich aller Bereiche außerhalb des Schulgebäudes wie z.B. auf dem Sportplatz?	1❑	2❑
…in Schulbussen oder anderen Verkehrsmitteln, die von den Schüler/-innen genutzt werden?	1❑	2❑
…in Schulveröffentlichen?	1❑	2❑
…bei Schulveranstaltungen, die gesponsert werden?	1❑	2❑

H. Hat Ihre Schule eine Raucherzone (z.B. einen Raum oder einen Bereich) für:

	ja	nein
Schüler/-innen	1❑	2❑
Mitarbeiter/-innen	1❑	2❑
Besucher/-innen	1❑	2❑

I. **Haben Sie im laufenden Schuljahr Schüler/-innen gesehen, die gegen die Rauchvorschriften Ihrer Schule verstoßen haben?**

- [1] ☐ nein
- [2] ☐ ja

J. **Gibt es an Ihrer Schule ein Vorgehen, um alle Schüler/-innen zu informieren über…**

	ja	nein
…die Vorschriften in Bezug auf das Rauchen bei Schüler/-innen?	1 ☐	2 ☐
…das weitere Vorgehen, wenn sie gegen die Regeln verstoßen?	1 ☐	2 ☐

K. **Welche der folgenden Methoden nutzt Ihre Schule, um Schüler/-innen über die Regelungen im Umgang mit dem Rauchen aufzuklären?**

	ja	nein
Eintrag in das Hausaufgabenheft von Schüler/-innen	1 ☐	2 ☐
Eintrag in das Klassenbuch	1 ☐	2 ☐
Bekanntmachung in einem Schulbrief	1 ☐	2 ☐
mündlich, in Gesprächen mit Schüler (z.B. Versammlungen, Schulrat)	1 ☐	2 ☐
Eintrag auf der/den Webseite(n) der Schule	1 ☐	2 ☐
Aushang in der Schule	1 ☐	2 ☐
per E-Mail	1 ☐	2 ☐

L. **Wie viele Schüler/-innen haben seit dem laufenden Schuljahr gegen die Rauchvorschriften Ihrer Schule verstoßen?**

- [1] ☐ keine
- [2] ☐ ein paar
- [3] ☐ einige
- [4] ☐ die meisten
- [5] ☐ alle

M. Was sind die Konsequenzen für Schüler/-innen, die im laufenden Schuljahr dabei erwischt wurden, wie sie gegen die Rauchvorschriften Ihrer Schule verstoßen haben?

Bitte alle zutreffenden Antworten ankreuzen!

- 1 ☐ keine
- 2 ☐ Erteilung einer Verwarnung (schriftlich/mündlich)
- 3 ☐ Benachrichtigung der Eltern
- 4 ☐ Benachrichtigung des Schulleiters/der Schulleiterin
- 5 ☐ Benachrichtigung des Beratungslehrers/der Beratungslehrerin
- 6 ☐ Die Teilnahme an einem Hilfe-, Bildungs- oder Raucherentwöhnungsprogramm wird angeregt, aber nicht gefordert.
- 7 ☐ Die Teilnahme an einem Hilfe-, Bildungs- oder Raucherentwöhnungsprogramm wird gefordert.
- 8 ☐ Zigaretten werden eingesammelt
- 9 ☐ Erteilung zusätzlicher Schularbeiten (schriftlich/mündlich)
- 10 ☐ Übertragung von Arbeiten auf dem Schulhof
- 11 ☐ andere Strafe
- 12 ☐ nachsitzen
- 13 ☐ Suspendierung
- 14 ☐ Schulverweis

N. Ist die Benutzung von elektronischen Zigaretten (E-Zigaretten) an Ihrer Schule erlaubt?

- 1 ☐ nein
- 2 ☐ ja

O. Gibt es an Ihrer Schule ein Vorgehen, um die Familien aller Schüler/-innen zu informieren über…

Kreuzen Sie nur eine Antwort pro Zeile an!

	ja, sie werden strikt befolgt	ja, sie werden aber nicht immer befolgt	ja, aber sie werden nie befolgt	nein
…die Vorschriften in Bezug auf das Rauchen bei Schüler/-innen?	1 ☐	2 ☐	3 ☐	4 ☐
…das weitere Vorgehen, wenn sie gegen die Regeln verstoßen?	1 ☐	2 ☐	3 ☐	4 ☐

P. **Gibt es an Ihrer Schule ein Vorgehen um Mitarbeiter/-innen zu informieren über…**

Kreuzen Sie nur eine Antwort pro Zeile an!

	ja, sie werden strikt befolgt	ja, sie werden aber nicht immer befolgt	ja, aber sie werden nie befolgt	nein
… die Vorschriften in Bezug auf das Rauchen bei Mitarbeiter/-innen?	1 ☐	2 ☐	3 ☐	4 ☐
… das weitere Vorgehen, wenn sie gegen die Regeln verstoßen?	1 ☐	2 ☐	3 ☐	4 ☐

Q. **Gibt es an Ihrer Schule ein Vorgehen um die Besucher/-innen zu informieren über…**

	ja	nein
…die Vorschriften in Bezug auf das Rauchen bei Besucher/-innen?	1 ☐	2 ☐
…das weitere Vorgehen, wenn sie gegen die Regeln verstoßen?	1 ☐	2 ☐

Gesundheitsförderung und Prävention an Ihrer Schule

Die folgenden Fragen beziehen sich auf Maßnahmen zur Gesundheitsförderung und Prävention an Ihrer Schule.

R. **Werden an Ihrer Schule Unterrichtsstunden zu den Konsequenzen des Rauchens durchgeführt?**

1 ☐ ja

2 ☐ nein

S. **Werden an Ihrer Schule Unterrichtsstunden speziell zur Gesundheitsförderung durchgeführt?**

1 ☐ ja

2 ☐ nein

T. **Für welche dieser Jahrgangsstufen bietet Ihre Schule eine Beratung zum Tabakkonsum an (z.B. pädagogische Maßnahmen)?**

Kreuzen Sie nur eine Antwort pro Zeile an!

		ja, es ist verpflichtend	ja, es wird angeboten	nein	weiß ich nicht
1.	Jahrgangsstufe 5	1❑	2❑	3❑	4❑
2.	Jahrgangsstufe 6	1❑	2❑	3❑	4❑
3.	Jahrgangsstufe 7	1❑	2❑	3❑	4❑
4.	Jahrgangsstufe 8	1❑	2❑	3❑	4❑
5.	Jahrgangsstufe 9	1❑	2❑	3❑	4❑
6.	Jahrgangsstufe 10	1❑	2❑	3❑	4❑
7.	Jahrgangsstufe 11	1❑	2❑	3❑	4❑
8.	Jahrgangsstufe 12	1❑	2❑	3❑	4❑

U. **Wie haben Sie in dem Curriculum Ihrer Schule ein Aufklärungsprogramm über das Rauchen integriert?**

Bitte alle zutreffenden Antworten ankreuzen

1❑ Es wird separat behandelt.

2❑ Es ist Teil des Schulgesundheitsprogramms.

3❑ Es wird im Zusammenhang mit anderen Themenbereichen behandelt (z.B. im Zusammenhang mit Biologie oder Religion/Ethik).

V. **Werden an Ihrer Schule unterhaltsame freiwillige oder nicht von Lehrer/-innen aufgezwungene Aktivitäten (z.B. Musik, körperliche Aktivitäten, etc.) für die Schüler/-innen in den Pausen als Alternative zum Rauchen angeboten?**

1❑ nein, überhaupt nicht

2❑ ja, aber sie werden kaum in Anspruch genommen

3❑ ja, sie werden umfassend in Anspruch genommen

Die folgenden Fragen beziehen sich auf Raucherentwöhnungsprogramme an Ihrer Schule.

W. **Bietet Ihre Schule Programme an, um Personen dabei zu unterstützen, das Rauchen aufzugeben (z.B. Gruppensitzungen, Selbsthilfematerialien, individuelle Beratung vom Schularzt)?**

1 ❑ ja, nur für Schüler/-innen

2 ❑ ja, nur für Lehrer/-innen und das Personal

3 ❑ ja, sowohl für Schüler-, Lehrer/-innen und das Personal

4 ❑ nein

X. **Hat Ihre Schule in den letzten sechs Monaten mit dem Gesundheitsamt, der Gesundheitsbehörde oder externen Fachleuten (z.B. Psychologen, Sozialarbeiter) an der Entwicklung oder Umsetzung von Maßnahmen gearbeitet, um zur Reduzierung des Rauchens zu motivieren oder ihnen beim Aufhören zu helfen?**

	ja	nein
Präventive Maßnahmen	1 ❑	2 ❑
Unterstützung beim Aufhören	1 ❑	2 ❑

Die nächsten Fragen beziehen sich auf verschiedene Optionen, die Ihre Schule anwendet, um Schüler/-innen, Familien und andere Personengruppen in Ihren Bestrebungen zur Aufklärung über den Tabakkonsum zu informieren.

Y. **Während der letzten sechs Monate waren…**

	ja	nein
…Schüler/-innen bei der Entwicklung, Vermittlung und Umsetzung von Maßnahmen zur Aufklärung über die Gefahren des Tabakkonsums beteiligt?	1 ❑	2 ❑
…die Familien der Schüler/-innen bei der Entwicklung, Vermittlung und Umsetzung von Maßnahmen zur Aufklärung über die Gefahren des Tabakkonsums beteiligt?	1 ❑	2 ❑
…Mitglieder der Gemeinde oder andere Personengruppen bei der Entwicklung, Vermittlung und Umsetzung von Maßnahmen zur Aufklärung über die Gefahren des Tabakkonsums beteiligt?	1 ❑	2 ❑

Z. **In welchem Umfang haben Sie Schulungen zur Prävention von Tabakkonsum in den letzten fünf Jahren erhalten?**

1 ❑ keine

2 ❑ mehr als einen ganzen Tag im Rahmen einer Fortbildung

3 ❑ einen ganzen Tag im Rahmen einer Fortbildung

4 ❑ weniger als einen ganzen Tag im Rahmen einer Fortbildung

5 ❑ Ich kann mich nicht erinnern.

**AA.**	**Inwieweit stimmen Sie den folgenden Aussagen zu?**<br>*Bitte machen Sie in jeder Zeile ein Kreuz!*				
		stimme überhaupt nicht zu	stimme nicht zu	stimme eher zu	stimme voll und ganz zu
1.	An Ihrer Schule ist ein Koordinator und/oder ein Komitee, welcher/s Aktivitäten zur Gesundheit und zum Wohlbefinden plant, umsetzt und koordiniert.	1 ❑	2 ❑	3 ❑	4 ❑
2.	Die Lehrzeit wird einem (oder einigen) Mitarbeiter(n) zugewiesen, um Maßnahmen zur Gesundheitsförderung zu planen, umzusetzen und zu koordinieren.	1 ❑	2 ❑	3 ❑	4 ❑
3.	Gesundheit und Wohlbefinden sind Teil der Schulstrategie bzw. des Entwicklungsplans der Schule.	1 ❑	2 ❑	3 ❑	4 ❑

Über Sie

Die folgenden Fragen betreffen Ihre Person.

BB. **Wie alt sind Sie?**

____

CC. **Ihr Geschlecht?**

1 ❑ weiblich

2 ❑ männlich

DD. **Was ist Ihre Hauptfunktion an dieser Schule?**

1 ❑ Schulleiter/-in

2 ❑ Konrektor/-in, stellvertretende(r) Schulleiter/-in

3 ❑ Aufsichtsperson

4 ❑ Lehrer/-in mit Zusatzausbildung/Funktion im Gesundheitsbereich/Sozialarbeiter/-in

5 ❑ sonstiges ____________________

EE. **Seit wann arbeiten Sie an dieser Schule?**

seit __________ Jahren

FF. **Rauchen Sie Zigaretten täglich, gelegentlich oder gar nicht?**

1 ☐ täglich

2 ☐ gelegentlich

3 ☐ gar nicht

Vielen Dank für Ihre Teilnahme an dieser Befragung!

Möchten Sie uns noch etwas mitteilen?

MARTIN-LUTHER-UNIVERSITÄT
HALLE-WITTENBERG

SILNER

MARTIN-LUTHER-UNIVERSITÄT
HALLE-WITTENBERG

Kurzfragebogen für Schulleitung

SILNER

Kurzdokumentation der teilnehmenden Schule

Bitte beantworten Sie uns ein paar Fragen zu Ihrer Schule.

Tragen Sie hier bitte den Namen und die Anschrift Ihrer Schule ein:

---

1. **Wie viele Schüler/-innen hat Ihre Schule?**

Anzahl Jungen _______

Anzahl Mädchen _______

2. **Wie viel Prozent der Schüler/-innen Ihrer Schule haben eine andere Muttersprache als Deutsch?**

Etwa __________%

3. **Wie viele Lehrkräfte gibt es an Ihrer Schule?**

Anzahl Lehrer/-innen _______

Anzahl Vertrauenslehrer/-in _______

4. **Wie viele Klassen pro Jahrgang gibt es durchschnittlich an Ihrer Schule?**

Anzahl __________

Wie viele Schüler/-innen pro Jahrgang gibt es durchschnittlich an Ihrer Schule?

Anzahl __________

5. **Wie wohlhabend ist die Gegend, in der sich Ihre Schule befindet?**

1 ❑ überhaupt nicht wohlhabend

2 ❑ nicht so wohlhabend

3 ❑ durchschnittlich

4 ❑ einigermaßen wohlhabend

5 ❑ sehr wohlhabend

6. **Wie wohlhabend sind die Familien Ihrer Schüler/-innen verglichen mit anderen Familien in der Stadt?**

- 1 ☐ sehr viel wohlhabender
- 2 ☐ viel wohlhabender
- 3 ☐ wohlhabender
- 4 ☐ ungefähr gleich
- 5 ☐ weniger wohlhabend
- 6 ☐ viel weniger wohlhabend
- 7 ☐ sehr viel weniger wohlhabend

Über Sie

Die folgenden Fragen betreffen Ihre Person.

BB. **Wie alt sind Sie?**

____

CC. **Ihr Geschlecht?**

- 1 ☐ weiblich
- 2 ☐ männlich

DD. **Was ist Ihre Hauptfunktion an dieser Schule?**

- 1 ☐ Schulleiter/-in
- 2 ☐ Konrektor/-in, stellvertretende(r) Schulleiter/-in
- 3 ☐ Aufsichtsperson
- 4 ☐ Lehrer/-in mit Zusatzausbildung/Funktion im Gesundheitsbereich/Sozialarbeiter/-in
- 5 ☐ sonstiges ____________________

EE. **Seit wann arbeiten Sie an dieser Schule?**

seit __________ Jahren

FF. **Rauchen Sie Zigaretten täglich, gelegentlich oder gar nicht?**

1 ☐ täglich

2 ☐ gelegentlich

3 ☐ gar nicht

Vielen Dank für Ihre Teilnahme an dieser Befragung!

Möchten Sie uns noch etwas mitteilen?
