# Supplementary material for: The association between school tobacco policies and the perceived smoking prevalence of adolescents
Source: Bundesgesundheitsblatt Gesundheitsforschung Gesundheitsschutz. 2020 Dec 7;64(1):91–101. [Article in German] doi: 10.1007/s00103-020-03261-1 (PMC7772164; doi:10.1007/s00103-020-03261-1)
Supplement: Supplementary file 2 [file 103_2020_3261_MOESM2_ESM.pdf]

Bundesgesundheitsblatt 1/2021

Elektronisches Zusatzmaterial zum Beitrag:

Der Zusammenhang zwischen schulischer Tabakkontrolle und der wahrgenommenen Raucherprävalenz Jugendlicher

Martin Mlinarić[1], Sebastian Günther[1], Irene Moor[1], Kristina Winter[1], Laura Hoffmann[1], Matthias Richter[1]

[1] Institut für Medizinische Soziologie, Medizinische Fakultät, Martin-Luther-Universität Halle-Wittenberg, Halle (Saale), Deutschland

Korrespondenzadresse

Dr. Martin Mlinarić
Institut für Medizinische Soziologie,
Medizinische Fakultät,
Martin-Luther-Universität Halle-Wittenberg,
Magdeburger Str. 8,
06112 Halle (Saale), Deutschland
martin.mlinaric@medizin.uni-halle.de

Inhalt:
Syntax zur Operationalisierung auf Basis von fünfzehn Fragen zur schulischen Tabakkontrolle

Syntax zur Operationalisierung auf Basis von fünfzehn Fragen zur schulischen Tabakkontrolle

```
*Staff*
use "C:\Users\mlinama\Desktop\Stata\Staff_2013_2017.dta"

*Missings*
*Recode Missings*
mvdecode _all, mv(888 = .)
mvdecode _all, mv(777 = .)
mvdecode _all, mv(444 = .)
mvdecode _all, mv(555 = .)

*Deutschland*
keep if country=="G"

*Tabakwaren 100m*
tab SQ3
codebook SQ3
clonevar Tabakwaren=SQ3
replace Tabakwaren= 0.5 if SQ3==1
replace Tabakwaren= 0 if SQ3==2
tab Tabakwaren

*Rauchverbot*
tab SQ5
codebook SQ5
clonevar Rauchverbot=SQ5
replace Rauchverbot= 0 if SQ5==1
replace Rauchverbot= 0.5 if SQ5==2
tab Rauchverbot

*Rauchvervot Verstoß letztes halbes Jahr*
tab SQ11
codebook SQ11
clonevar Verstoß=SQ11
replace Verstoß= 0 if SQ11==2
replace Verstoß= 0.5 if SQ11==1
tab Verstoß

*SF school building*
codebook SQ7_1sta
gen sta_building=SQ7_1sta
replace sta_building = 0.5 if SQ7_1sta==2
replace sta_building = 0 if SQ7_1sta==1
tab sta_building

codebook SQ7_1stu
gen stu_building=SQ7_1stu
replace stu_building = 0.5 if SQ7_1stu==2
replace stu_building = 0 if SQ7_1stu==1
```

```
tab stu_building

*SF school premises*
codebook SQ7_2sta
gen sta_schoolpremises=SQ7_2sta
replace sta_schoolpremises=0.5 if SQ7_2sta==2
replace sta_schoolpremises=0 if SQ7_2sta==1
tab sta_schoolpremises

codebook SQ7_2stu
gen stu_schoolpremises=SQ7_2stu
replace stu_schoolpremises=0.5 if SQ7_2stu==2
replace stu_schoolpremises=0 if SQ7_2stu==1
tab stu_schoolpremises

*SF outside*
codebook SQ7_4sta
gen sta_outside=SQ7_4sta
replace sta_outside=0.5 if SQ7_4sta==2
replace sta_outside=0 if SQ7_4sta==1
tab sta_outside

codebook SQ7_4stu
gen stu_outside=SQ7_4stu
replace stu_outside=0.5 if SQ7_4stu==2
replace stu_outside=0 if SQ7_4stu==1
tab stu_outside

*Smoking spot at school*
codebook SQ10_1
gen stu_smokingspot=SQ10_1
replace stu_smokingspot=0.5 if SQ10_1==1
replace stu_smokingspot=0 if SQ10_1==2
tab stu_smokingspot

codebook SQ10_2
gen sta_smokingspot=SQ10_2
replace sta_smokingspot=0.5 if SQ10_2==1
replace sta_smokingspot=0 if SQ10_2==2
tab sta_smokingspot

*Curriculum*
codebook SQ19 SN3
gen curriculum=SQ19
replace curriculum=0 if SQ19==1
replace curriculum=0.5 if SQ19==2
tab curriculum

tab1 Tabakwaren Rauchverbot sta_building stu_building sta_schoolpremises
stu_schoolpremises sta_outside stu_outside sta_smokingspot stu_smokingspot curriculum
quietly logistic Tabakwaren Rauchverbot Verstoß sta_building stu_building sta_schoolpremises
stu_schoolpremises sta_outside stu_outside sta_smokingspot stu_smokingspot curriculum
```

```
gen sample = e(sample)

*UV STaff*

alpha Tabakwaren Rauchverbot Verstoß sta_building stu_building sta_schoolpremises
stu_schoolpremises sta_outside stu_outside sta_smokingspot stu_smokingspot curriculum,
min(11) item gen(STP_staff_avg)
summarize STP_staff_avg, detail

alpha Tabakwaren Rauchverbot Verstoß sta_building stu_building sta_schoolpremises
stu_schoolpremises sta_outside stu_outside sta_smokingspot stu_smokingspot curriculum
correlate Tabakwaren Rauchverbot Verstoß sta_building stu_building sta_schoolpremises
stu_schoolpremises sta_outside stu_outside sta_smokingspot stu_smokingspot curriculum
gen
STP_staff_sum=Tabakwaren+Rauchverbot+Verstoß+sta_building+stu_building+sta_schoolpre
mises+stu_schoolpremises+sta_outside+stu_outside+sta_smokingspot+stu_smokingspot+curri
culum
tab STP_staff_sum
summarize STP_staff_sum if sample==1, detail
by Year, sort: sum STP_staff_sum if sample==1, detail
hist STP_staff_sum if sample==1
clonevar Schulpersonal=STP_staff_sum
histogram Schulpersonal if sample==1

by Year id_school, sort : summarize STP_staff_sum, detail
by Year school_number, sort : summarize STP_staff_sum, detail
by Year id_school, sort : summarize STP_staff_avg, detail
by Year school_number, sort : summarize STP_staff_avg, detail

clear
*Students*
use "C:\Users\mlinama\Desktop\Stata\SILNE_2013_2017.dta"

*Deutschland*
keep if country_rec==276
*Exclude non-participants*
codebook status
keep if status==888

*Missings*
*Recode Missings*
mvdecode _all, mv(888 = .)
mvdecode _all, mv(777 = .)
mvdecode _all, mv(444 = .)
mvdecode _all, mv(555 = .)

*german schools*
tab1 id_school school_number
destring id_school, generate(Schule)
destring school_number, generate(Schulnummer)

*AV: Q36*
```

```
summarize Q36, detail
tabulate Q36, nolabel
histogram Q36
codebook Q36

clonevar Rauchprävalenz=Q36
histogram Rauchprävalenz
sktest Rauchprävalenz

*Staff STPs*
gen staff_STP=0
label var staff_STP "Schulische Tabakkontrolle Lehrer"
label define staff_STP 1 "schwach" 2 "mittel" 3 "stark"
label values staff_STP staff_STP
replace staff_STP = 2 if Schule==51
replace staff_STP = 1 if Schule==52
replace staff_STP = 2 if Schule==53
replace staff_STP = 3 if Schule==54
replace staff_STP = 1 if Schule==55
replace staff_STP = 3 if Schule==56
replace staff_STP = 2 if Schule==57
replace staff_STP = 2 if Schule==58
replace staff_STP = 3 if Schule==59
replace staff_STP = 3 if Schule==60 & Year==2013
replace staff_STP = 2 if Schule==60 & Year==2016
replace staff_STP = 1 if Schule==61
replace staff_STP = 1 if Schule==62
replace staff_STP = 2 if Schule==63
replace staff_STP = 1 if Schule==50
replace staff_STP = 2 if Schule==90
replace staff_STP = 1 if Schule==91
replace staff_STP = 2 if Schule==92
replace staff_STP = 1 if Schule==93
replace staff_STP = 2 if Schule==94
replace staff_STP = 3 if Schule==95
replace staff_STP = 3 if Schule==96
replace staff_STP = 2 if Schule==97
replace staff_STP = 2 if Schule==98
replace staff_STP = 3 if Schule==99
tab staff_STP

gen staff_STP_sum=0
label var staff_STP_sum "Tabakkontrolle Lehrer"
replace staff_STP_sum = 4.7 if Schule==51
replace staff_STP_sum = 4.5 if Schule==52
replace staff_STP_sum = 4.8 if Schule==53
replace staff_STP_sum = 4.9 if Schule==54
replace staff_STP_sum = 4.3 if Schule==55
replace staff_STP_sum = 4.9 if Schule==56
replace staff_STP_sum = 4.6 if Schule==57
replace staff_STP_sum = 4.8 if Schule==58
```

```
replace staff_STP_sum = 5.5 if Schule==59
replace staff_STP_sum = 5.0 if Schule==60 & Year==2013
replace staff_STP_sum = 4.8 if Schule==60 & Year==2016
replace staff_STP_sum = 4.4 if Schule==61
replace staff_STP_sum = 4.0 if Schule==62
replace staff_STP_sum = 4.8 if Schule==63
replace staff_STP_sum = 4.5 if Schule==50
replace staff_STP_sum = 4.8 if Schule==90
replace staff_STP_sum = 3.4 if Schule==91
replace staff_STP_sum = 4.6 if Schule==92
replace staff_STP_sum = 4.4 if Schule==93
replace staff_STP_sum = 4.8 if Schule==94
replace staff_STP_sum = 5.0 if Schule==95
replace staff_STP_sum = 5.0 if Schule==96
replace staff_STP_sum = 4.7 if Schule==97
replace staff_STP_sum = 4.7 if Schule==98
replace staff_STP_sum = 5.5 if Schule==99

summarize staff_STP_sum, detail
tab staff_STP
hist staff_STP_sum

regress Rauchprävalenz ib(last).staff_STP staff_STP_sum, vce(cluster Schule)
tab2 Rauchprävalenz staff_STP
tab2 Rauchprävalenz staff_STP_sum

*UV: STPs*
tab staff_STP
tab1 Q45 S61 Q32_1 Q32_2
*Wahrnehmung Schüler Rauchverbot 2017*
clonevar STP2017 = Q45
codebook STP2017
replace STP2017 = 2 if Q45==1
replace STP2017 = 1 if Q45==2
replace STP2017 = 0.5 if Q45==3
replace STP2017 = 0 if Q45==4
replace STP2017 = 0 if Q45==5
label drop Q45
tab STP2017
*Wahrnehmung Schüler Rauchverbot 2013*
clonevar STP2013 = S61
codebook STP2013
replace STP2013 = 0 if S61==1
replace STP2013 = 0.5 if S61==2
replace STP2013 = 1 if S61==3
replace STP2013 = 2 if S61==4
replace STP2013 = 0 if S61==5
label drop S61
tab STP2013

tab1 STP2013 STP2017
```

```
gen STP_SILNER = STP2013
replace STP_SILNER = 2 if STP2013==. & STP2017==2
replace STP_SILNER = 1 if STP2013==. & STP2017==1
replace STP_SILNER = 0.5 if STP2013==. & STP2017==0.5
replace STP_SILNER = 0 if STP2013==. & STP2017==0

tab STP_SILNER

*Sichtbarkeit*
tab1 Q32_1 Q32_2
codebook Q32_1 Q32_2

*Sichtbarkeit Schüler*
clonevar vis_stu = Q32_1
codebook vis_stu
replace vis_stu = 2 if Q32_1==1
replace vis_stu = 1 if Q32_1==2
replace vis_stu = 0.5 if Q32_1==3
replace vis_stu = 0 if Q32_1==4
label drop Q32_1
tab1 vis_stu Q32_1

*Sichtbarkeit Lehrer*
clonevar vis_sta = Q32_2
codebook vis_sta
replace vis_sta = 2 if Q32_2==1
replace vis_sta = 1 if Q32_2==2
replace vis_sta = 0.5 if Q32_2==3
replace vis_sta = 0 if Q32_2==4
label drop Q32_2
tab1 vis_sta Q32_2

alpha STP_SILNER vis_stu vis_sta

gen STP_stu_sum=STP_SILNER+vis_stu+vis_sta
label var staff_STP_sum "Tabakkontrolle Schüler"
tab STP_stu_sum
summarize STP_stu_sum, detail
clonevar Schüler=STP_stu_sum
histogram Schüler

by Year Schule, sort : summarize STP_stu_sum, detail

grmeanby Schule, summarize(STP_stu_sum)
grmeanby Schule, summarize(STP_stu_sum) median

grmeanby Schule, summarize(staff_STP_sum)
grmeanby Schule, summarize(staff_STP_sum) median

gen stu_STP=0
label var stu_STP "Schulische Tabakkontrolle Lehrer"
label define stu_STP 1 "schwach" 2 "mittel" 3 "stark"
```

```
label values stu_STP stu_STP
replace stu_STP = 2 if Schule==51
replace stu_STP = 2 if Schule==52
replace stu_STP = 1 if Schule==53
replace stu_STP = 2 if Schule==54
replace stu_STP = 1 if Schule==55
replace stu_STP = 3 if Schule==56
replace stu_STP = 1 if Schule==57
replace stu_STP = 2 if Schule==58
replace stu_STP = 2 if Schule==59
replace stu_STP = 2 if Schule==60 & Year==2013
replace stu_STP = 3 if Schule==60 & Year==2016
replace stu_STP = 1 if Schule==61
replace stu_STP = 2 if Schule==62
replace stu_STP = 2 if Schule==63
replace stu_STP = 3 if Schule==50
replace stu_STP = 2 if Schule==90
replace stu_STP = 2 if Schule==91
replace stu_STP = 2 if Schule==92
replace stu_STP = 3 if Schule==93
replace stu_STP = 2 if Schule==94
replace stu_STP = 3 if Schule==95
replace stu_STP = 3 if Schule==96
replace stu_STP = 2 if Schule==97
replace stu_STP = 3 if Schule==98
replace stu_STP = 3 if Schule==99
tab stu_STP

regress Rauchprävalenz ib(last).stu_STP STP_stu_sum, vce(cluster Schule)
tab2 Rauchprävalenz stu_STP
tab2 Rauchprävalenz STP_stu_sum

*Correlate teacher & students*
correlate staff_STP_sum STP_stu_sum
```
